# Quinolone Signals Related to *Pseudomonas* Quinolone Signal-Quorum Sensing Inhibits the Predatory Activity of *Bdellovibrio bacteriovorus*

**DOI:** 10.3389/fmicb.2021.722579

**Published:** 2021-09-10

**Authors:** Yuki Hoshiko, Yoshito Nishiyama, Tae Moriya, Kiwao Kadokami, Luis Esaú López-Jácome, Ryutaro Hirano, Rodolfo García-Contreras, Toshinari Maeda

**Affiliations:** ^1^Department of Biological Functions Engineering, Graduate School of Life Science and Systems Engineering, Kyushu Institute of Technology, Kitakyushu, Japan; ^2^Institute of Environmental Science and Technology, The University of Kitakyushu, Kitakyushu, Japan; ^3^Department of Microbiology and Parasitology, Faculty of Medicine, UNAM, Mexico City, Mexico; ^4^Laboratory of Infectology, National Institute of Rehabilitation Luis Guillermo Ibarra Ibarra, Mexico City, Mexico

**Keywords:** inhibition factors, *Escherichia coli*, quorum sensing, *Pseudomonas* quinolone signal, *Pseudomonas aeruginosa*, *Bdellovibrio bacteriovorus*, predatory bacteria

## Abstract

*Bdellovibrio bacteriovorus* is one of the predatory bacteria; therefore, it can act as a novel “living antibiotic,” unlike the current antibiotics. Here the predation of *Escherichia coli* by *B. bacteriovorus* was inhibited in the presence of *Pseudomonas aeruginosa*. This study investigated whether *P. aeruginosa*-induced predation inhibition is associated with bacterial quorum sensing (QS). Each *las*, *rhl*, or *pqs* QS mutant in *P. aeruginosa* was used to check the predatory activity of *E. coli* cells using *B. bacteriovorus*. As a result, the predatory activity of *B. bacteriovorus* increased in a mutant *pqs* QS system, whereas wild-type PA14 inhibited the predatory activity. Moreover, the addition of 4-hydroxy-2-heptylquinoline (HHQ) or the analog triggered the low predatory activity of *B. bacteriovorus* and killed *B. bacteriovorus* cells. Therefore, a defensive action of *P. aeruginosa* against *B. bacteriovorus* is activated by the *pqs* QS system, which produces some quinolone compounds such as HHQ.

## Introduction

*Bdellovibrio bacteriovorus* is a small-sized Gram-negative bacterium belonging to the group *Bdellovibrio* and like organisms (BALOs). It is one of the obligate predatory bacteria which prey another Gram-negative bacterium (Stolp and Starr, 1963). Although *B. bacteriovorus* cannot prey upon Gram-positive bacterial species, it is known to be able to degrade the biofilm formed by Gram-negative bacteria and Gram-positive ones (Kadouri and O’Toole, 2005; Dashiff et al., 2011; Im et al., 2018). *B. bacteriovorus* also has a competitive inhibition against *Staphylococcus aureus* (Im et al., 2018). BALOs exist in a diverse environment such as waste sewage sludge (Koval et al., 2013), river (Hobley et al., 2012), sea (Pineiro et al., 2007; Chen et al., 2015; Tang et al., 2020), and various habitats on the ground (Jurkevitch et al., 2000). *B. bacteriovorus* usually grows through the invasion, digestion, and lysis of other Gram-negative bacteria as host cells; then, it again swims to find other new host cells, predate, and replicate further (Pérez et al., 2016). In the predation for Gram-negative bacteria, *B. bacteriovorus* takes 2–4 h to complete all the processes, including the attachment using type IVa pili (Evans et al., 2007; Mahmoud and Koval, 2010), the modification of peptidoglycan of host cells (Stolp and Starr, 1965; Kuru et al., 2017), the digestion, and the lysis, which some enzymes can handle as obtained from *B. bacteriovorus* (Thomashow and Rittenberg, 1978a,b; Lerner et al., 2012). Hence, *B. bacteriovorus* has the potential to be applied in many fields—for example, *B. bacteriovorus* may have a certain potential to kill multidrug-resistant bacteria (Iebba et al., 2014), remove harmful bacteria on food (Fratamico and Cooke, 1996), decrease waste sewage sludge (Semblante et al., 2017), and disperse biofilm on the pipes in wastewater treatment plants (Feng et al., 2016). To date, *B. bacteriovorus* is expected to be available for cystic fibrosis patients with chronic infections (Sockett and Lambert, 2004; Shatzkes et al., 2015) caused by *Pseudomonas aeruginosa* which is known as one of the critical strains of multidrug-resistant bacteria because *B. bacteriovorus* was discovered to prey on *P. aeruginosa* by some researchers (Stolp and Starr, 1963; Shanks et al., 2013; Iebba et al., 2014). However, other researchers also reported that *P. aeruginosa* is not preyed upon by *B. bacteriovorus* (Mukherjee et al., 2015). Thus, the predatory performance of *B. bacteriovorus* to *P. aeruginosa* strains is inconsistent; however, the reason is still unknown.

Most bacteria in nature do not live independently but communicate with each other through extracellular signal molecules to control their collective behavior. These signal molecules are mechanisms released into the environment and respond after they are taken up by the surrounding cells (Fuqua et al., 2001; Miller and Bassler, 2001; Withers et al., 2001). Quorum sensing (QS), one of the most essential systems for regulating bacterial functions, is a typical communication system used by many bacterial species to activate or repress a large number of genes, which form the QS regulon. Changes in their expression levels are achieved once the signal molecules reach a given threshold (Fuqua et al., 1994). It controls bacterial functions such as biofilm formation, resistance to acid stress, motility, competence, and morphological modifications (Huang et al., 2016; Johansen and Jespersen, 2017; de Almeida et al., 2018). Gram-negative bacteria use small diffusible molecules called autoinducers such as indole (Lee et al., 2015), acyl-homoserine lactones, autoinducer-2 (Galloway et al., 2011), and alkyl-quinolone (Pesci et al., 1999) among others—for example, *P. aeruginosa* also regulates the gene expression of some virulence factors by using four QS systems directly or indirectly: *las* regulation system, *rhl* regulation system, *pqs* regulation system, and *iqs* regulation system (Winson et al., 1995; Latifi et al., 1996; Pesci et al., 1997, 1999; McKnight et al., 2000; Gallagher et al., 2002; Kiratisin et al., 2002; Déziel et al., 2004; Xiao et al., 2006; Lee et al., 2013). *P. aeruginosa* uses 3-oxo-C_12_-homoserine lactone for the *las* QS system, C_4_-homoserine lactone for the *rhl* QS system, and integrated QS signal [IQS; 2-(2-hydroxyphenyl)-thiazole-4-carbaldehyde]. In particular, the *pqs* QS system is known as the most complicated one among the QS systems of *P. aeruginosa* since the system has three kinds of quinolone compounds: *Pseudomonas* quinolone signals [PQS; 2-heptyl-3-hydroxy-4(1H)-quinolone], 4-hydroxy-2-heptylquinolone (HHQ), and 2-heptyl-4-quinolinol-1-oxide (HQNO). Thus, all the QS signal molecules have the function of promoting or inhibiting other bacteria, fungi, protozoa, and nematodes (Fuqua et al., 1994; Burgess et al., 1999; Dubuis et al., 2007). Changes in the expression of autoinducer and QS-controlled compounds can also affect not only bacterial community dynamics but also host responses during infection. It has been reported that various QS regulatory molecules, such as acyl-homoserine lactones, quinolones, and phenazines, can interact with host cells and thereby affect various responses, including immune regulation (Liu et al., 2015). However, *E. coli* strains, including *E. coli* O157:H7 (Page et al., 2015), use indole as one of the signal molecules (Wang et al., 2001), and Dwidar et al. (2015) investigated that a high concentration (2 mM) of indole inhibits the predation of *E. coli* by *B. bacteriovorus*. Since 0.25–1.1 mM indole was detected in human feces (Karlin et al., 1985; Zuccato et al., 1993), indole may help *E. coli* to inactivate the predatory activity of *B. bacteriovorus* if they grow together. Thus, we hypothesized that the QS systems of Gram-negative bacteria might influence the *B. bacteriovorus* cells negatively.

Although the effect of indole on the predatory activity of *B. bacteriovorus* was investigated, there are no reports on how other QS molecules of Gram-negative bacteria influence the activity of *B. bacteriovorus*. In this study, the relationship between the QS systems of *P. aeruginosa* and the predatory activities of *B. bacteriovorus* was investigated.

## Materials and Methods

### Chemicals

The following compounds, 2-heptyl-3-hydroxy-4(1H)-quinolone (PQS; *Pseudomonas* quinolone signal), 4-hydroxy-2-heptylquinolone (HHQ), and 2-amino-6-chlorobenzoic acid (CABA), which is a *pqs* QS inhibitor, were purchased from Sigma-Aldrich Japan (Tokyo, Japan), and 2-heptyl-4-quinolinol-1-oxide (HQNO) was purchased from Cayman Chemical Company (Michigan, United States). As one of the quinolone analogs, nalidixic acid was also purchased from FUJIFILM Wako Pure Chemical Corporation, Ltd. (Osaka, Japan). PQS and HHQ were stocked in dimethyl sulfoxide (DMSO) to make an 80-mM standard solution. HQNO, nalidixic acid, and CABA were stocked in DMSO to be 8-mM, 16-mM, and 1.5-M stock solutions, respectively.

### Bacterial Strains

*B. bacteriovorus* 109J (ATCC 43826), used as a predatory bacterium in this study, was purchased from the ATCC culture collection. The glycerol stock of *B. bacteriovorus* cells was made by mixing a cell culture of *B. bacteriovorus* and an equal volume of 2 × stock solution consisting of 10 g of yeast extract, 100 ml DMSO, 100 ml glycerol, 312 ml of 0.2 M NaH_2_PO_4_, and 488 ml of 0.2 M Na_2_HPO_4_ in 1 liter of the total stock solution, and the mixture was stored at −70°C. The *Escherichia coli* strains and *P. aeruginosa* strains used in this study are listed in Table 1.

**TABLE 1:** Strains used as host cells in this study.

**Strains**	**Genotype**	**Sources**
*Escherichia coli* K-12 BW25113	*lacI*^*q*^*rrnB_T__14_*Δ*lacZ_WJ_*_16_*hsdR514*Δ*araBAD_AH__33_*Δ*rhaBAD_LD__78_*; parental strain for the Keio collection	Datsenko and Wanner, 2000
*E. coli* K-12 BW25113 *tnaA*	K-12 BW25113 Δ*tnaA* Ω Km^*R*^	Baba et al., 2006
*Pseudomonas aeruginosa* PA14	*P. aeruginosa* wild type	Liberati et al., 2006
*P. aeruginosa pqsA*	PA14 *pqsA* Ω *Mar2xT7*, Gm^*R*^	Liberati et al., 2006
*P. aeruginosa pqsH*	PA14 *pqsH* Ω *Mar2xT7*, Gm^*R*^	Liberati et al., 2006
*P. aeruginosa lasI*	PA14 *lasI* Ω *Mar2xT7*, Gm^*R*^	Liberati et al., 2006
*P. aeruginosa rhlI*	PA14 *rhlI* Ω *Mar2xT7*, Gm^*R*^	Liberati et al., 2006

*Km^R^, kanamycin resistance; Gm^*R*^, gentamicin resistance.*

### Growth Condition and Predation Assay

These strains were initially streaked on Luria Bertani (LB) (Sambrook et al., 1989) agar plates and incubated overnight at 37°C. Then, a single colony of each strain was used for inoculation. For the cell preparation of prey cells, a single colony of *E. coli* and *P. aeruginosa* strains was inoculated into 100 ml of LB medium and aerobically incubated overnight at 37°C and 120 rpm. Mainly *E. coli* cells in the log phase (8–12 h) were used. The overnight culture was centrifuged for 10 min at 7,000 rpm and 4°C to wash the cell pellet using HEPES-based buffer, including 25 mM HEPES and 2 mM CaCl_2_ (the pH was adjusted to 7.6 using 5 M NaOH). Then, each washed pellet was suspended and adjusted at ∼1.5 or two of OD_600_ by the same HEPES buffer. The final cell suspensions were used for propagating *B. bacteriovorus* cells (when *E. coli* BW25113 was used) and for the predation assays in the experiments. For the predation assay using the double-layer method, as mentioned above, the top agar (3 ml) consisting of *B. bacteriovorus* culture (100 μl) and *E. coli* overnight culture (100 μl) was first poured to the bottom agar plate. After the agar has hardened, 2 μl of bacterial overnight culture and/or 2 μl of standard chemical solution was put on the center of each plate, and these plates were incubated at 30°C for 2–3 days. An inhibition zone (the area where *E. coli* cells were grown) was measured with a ruler at three points per plate. Three independent experiments were carried out.

### Preparation of *B. bacteriovorus* Cells and Double-Layer Method

Initially, the purity of *B. bacteriovorus* stock was confirmed by checking the plaque formation in a double-layer method in which 3 ml nutrient broth (NB) top agar (kept warm at 50°C; 8 g/l NB, 0.286 g/l MgCl_2_, 0.222 g/l CaCl_2_, and 8 g/l agar in pure water) mixed with 100 μl *B. bacteriovorus* culture and 100 μl of *E. coli* growth culture (at 37°C and 120 rpm for 10 h) was poured to NB agar bottom plates consisting of 10 g/l agar concentration and incubated at 30°C for around a week. A piece of top agar with the plaque of *B. bacteriovorus* was sliced at 1 cm^2^ and transferred under sterile conditions to the *E. coli* cell suspension (60 ml). The mixture was incubated at 37°C and 120 rpm for 2–3 days until the cell turbidity, mainly derived from *E. coli* cells, drastically decreased. Then, a pure cell culture of *B. bacteriovorus* was obtained by removing *E. coli* cells from the culture with a 0.45-μm-pore-size membrane filter. The filtrate was used as the cell culture of *B. bacteriovorus* for all the experiments in this study.

### Viability Tests of Prey Cells in the Presence of *B. bacteriovorus*

*E. coli* and *P. aeruginosa* cell suspensions, as mentioned above, were concentrated to OD_600_ of 2.0. The *E. coli* cell suspensions (5 ml) were incubated with or without the inoculation of *B. bacteriovorus* cell culture (167 μl) (multiplicity of infection, MOI = 0.01–0.1). The MOI was calculated by dividing the viable count of *B. bacteriovorus* in a 167-μl cell culture by the viable count of *E. coli* in 5-ml cell suspension at ∼2 of the cell turbidity at OD_600_. *B. bacteriovorus* cell culture (167 μl) was also inoculated in a mixture of 2.5 ml *E. coli* and *P. aeruginosa* cell suspension. As a control, without *B. bacteriovorus*, the sample with only *E. coli* and *P. aeruginosa* cell suspension was also prepared. These mixtures were incubated at 30°C and 120 rpm for a week. Then, an appropriate serial dilution of tester samples was prepared by using 0.85% sterile NaCl solution. To evaluate the survival of the prey cells, serial dilution was spread on LB agar plates and incubated at 37°C under aerobic conditions overnight. Then, the colonies grown on each plate were counted. The distinction between *E. coli* colonies and *P. aeruginosa* colonies was determined visually based on the shape, size, and color of the colonies. Here the plates with pure cultures of *E. coli* and *P. aeruginosa*, respectively, were prepared as standards. Two or three independent experiments were carried out.

### Pyocyanin Extraction

For pyocyanin extraction, a single colony of *P. aeruginosa* PA14 was inoculated in 5 ml of LB medium and incubated overnight at 37°C and 120 rpm. The overnight culture (1.83 ml) was inoculated in 0.25% casein-M9 medium (100 ml) to be 0.05 of the cell turbidity at OD_600_ (2.5 g casein, 6.0 g Na_2_HPO_4_, 3.0 g KH_2_PO_4_, 1.0 g NH_4_Cl, 0.5 g NaCl, 2 ml of 1 M MgSO_4_, and 0.1 ml of 1 M CaCl_2_ in 1 liter of water; the pH was adjusted to 7 using 5 M NaOH) and incubated for 24 h at 37°C and 180 rpm. Pyocyanin was extracted by using chloroform and 0.2 M HCl (Essar et al., 1990) from the overnight culture (100 ml) of *P. aeruginosa* PA14. The concentration of pyocyanin extracted was determined by measuring the absorbance at 520 nm using a V-530 spectrophotometer (JASCO Corporation, Tokyo, Japan). The molar absorptivity of acidic pyocyanin (ε = 2.46 mM^–1^ cm^–1^) (O’Malley et al., 2004) was used for the calculation of the concentration. The acidic pyocyanin solution was finally adjusted to pH ∼7 by adding 5 M NaOH and stocked at a concentration of 6.63 mM. The stock solution was filtered with a 0.20-μm membrane filter.

### Viability Tests of Predatory Cells in the Presence of Quinolone Compounds

Each solution of quinolone compounds was mixed with *B. bacteriovorus* cell culture (0.5 ml) and HEPES buffer (4.5 ml) to be at a concentration of 80 μM. As a control including the only solvent, 2 μl DMSO was added to the *B. bacteriovorus* mixture in HEPES buffer without any chemical compound. The mixture was incubated at 30°C and 120 rpm. The mixture was collected in 0, 3, and 6 days to measure the number of viable predatory cells. In evaluating the viability of predatory cells, the double-layer method mentioned above was conducted. An appropriate serial dilution of the *B. bacteriovorus* cell culture was used to count the number (30–300) of plaques formed on the agar plates after incubation at 30°C for around a week. Two independent experiments were carried out. For the metabolic activity assay, 180 μl HEPES buffer (*pH* = 7.6) with 80 μM PQS, HHQ, HQNO, and nalidixic acid or 0.1% DMSO as the control condition, 10 μl of *B. bacteriovorus* culture (∼10^6^ cells), and 10 μl of color reagent (WST solution: electron mediator reagent = 9:1) purchased from Kishida Chemical Co., Ltd. (Osaka, Japan) were mixed in a well of a 96-well plate. The plate was incubated at 30°C, and the microplate reader measured the aerobic conditions and the absorbance at 450 nm with time (every 12 h up to 48 h). Three independent experiments were carried out.

### Quinolone Compound Extraction and Quantification

*P. aeruginosa* PA14 was cultured in LB medium with 0, 0.75, 1.5, and 3 mM CABA (solvent: DMSO, stock concentration: 1.5 M) by incubation at 37°C and 120 rpm for 10 h. The 5-ml culture was centrifuged at 13,000 rpm for 5 min and filtered using a 0.2-μm-pore-size filter. The supernatant (4 ml) was mixed with 4 ml ethyl acetate (Lépine et al., 2004; Lesic et al., 2007) to extract quinolone compounds. The organic solvent was further evaporated, and the dried fraction of quinolone compounds dissolved in 10 μl DMSO. The final DMSO solution was used to test the growth of *B. bacteriovorus*. Then, 2 μl of DMSO solution containing quinolone compounds or 100% DMSO as a control was put on the center of each plate with the cells of *B. bacteriovorus* and *E. coli* in the top agar as mentioned above. Then, these plates were incubated at 30°C for 3–4 days.

In accordance with Supplementary Tables 1, 2, LC-QTOF-MS was performed to quantify each quinolone compound in each fraction. Basically, the conditions and methods were followed as written by Kadokami and Ueno (2019). The ion transitions of each quinolone compound are shown in Supplementary Table 3. The calibrations of the concentrations prepared were 1, 5, 10, 50, 100, 500, and 1,000 ng/l of each chemical dissolved in methanol. Three independent experiments were carried out.

### Isolation of *Bdellovibrio*-Like Bacteria From River Water

River water was collected from Onga River, Kitakyushu, Japan, and an aliquot of the river water (100 ml) was mixed with *E. coli* cells suspended with HEPES buffer at ∼2 of cell turbidity at OD_600_ (100 ml) to aggregate *B. bacteriovorus* from the river. The mixture was incubated at 30°C and 180 rpm for 2 days. The enriched culture was filtered by a 0.45-μm-pore-size filter, and the filtrate was used for the double-layer method as mentioned above to obtain a single plaque of *Bdellovibrio*-like bacteria. A piece of a top agar plate with the plaque was sliced and added to the *E. coli* cell suspension until the cell turbidity (OD_600_) decreased (for 3–4 days). A pure culture of a *Bdellovibrio*-like bacterium was obtained by repeating this step three times according to the method of Hobley et al. (2012). Three independent experiments were carried out.

### Indole Production Assay

*E. coli* culture in LB medium was incubated at 30°C and 120 rpm, and the cell turbidity was measured at 600 nm after 24 h. The *E. coli* supernatant (0.5 ml) of the 24-h culture was reacted to 0.2 ml of Kovac’s reagent (mixed with 5 g of *p*-dimethylamino-benzaldehyde, 25 ml HCl, and 75 ml pentanol). The organic layer (0.1 ml), Kovac’s reagent, was mixed with 0.9 ml HCl-amyl alcohol mixture (30 ml HCl and 90 ml pentanol). The absorbance at 540 nm was measured using a V-530 spectrophotometer (JASCO Corporation, Tokyo, Japan) to determine the indole concentration (Kawamura-Sato et al., 1999). Three independent experiments were carried out.

### Statistical Analysis

Compared with the control without any test reagent, each chemical sample was calculated using the means from at least duplicate data (*n* = 2 or 3). Graphics were created using Microsoft Office. According to the Shapiro–Wilk test, each data was normally distributed. Therefore, differences of some results were determined by one-way ANOVA, followed by Tukey’s test at a significance level of *p* < 0.05.

## Results

### Effect of *P. aeruginosa* on the Predation of *E. coli* by *B. bacteriovorus*

**FIGURE 1:**
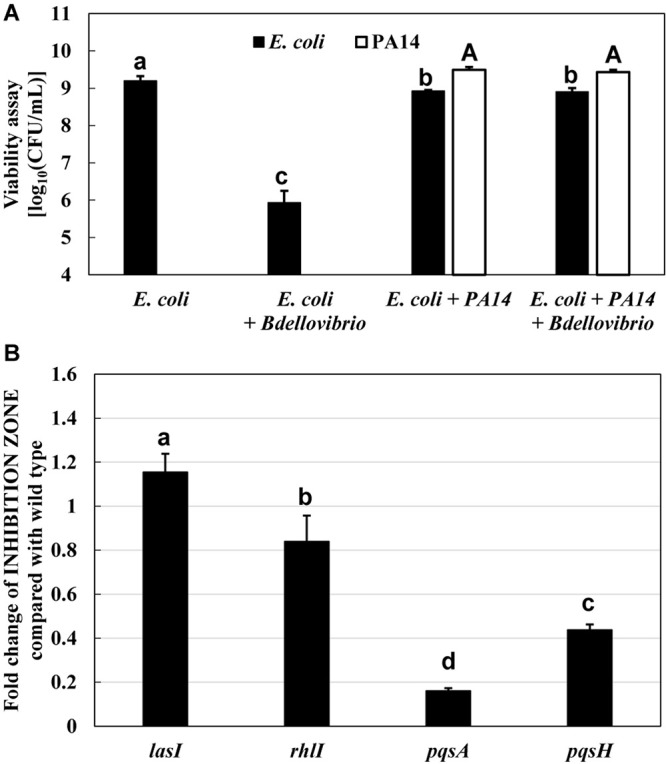
Inhibition of the predatory activity of *Bdellovibrio bacteriovorus* in the presence of *Pseudomonas aeruginosa*. **(A)** Dynamics of viable cells of *Escherichia coli* or/and *P. aeruginosa* PA14 with or without *B. bacteriovorus* 109J. Each cell suspension (initial cell turbidity was adjusted to be ∼2 at OD_600_) in HEPES buffer was incubated for 72 h at 30°C and 120 rpm under aerobic condition (*n* = 2). **(B)** Effect of *P. aeruginosa* quorum-sensing mutants (*las*, *rhl*, and *pqs*) on the predation plate assays using a mixture of *B. bacteriovorus* 109J and *E. coli* BW25113. In the plate assays using *E. coli* BW25113 as a prey cell, the area of the inhibition zone was compared in the presence of each *P. aeruginosa* strain (*P. aeruginosa* wild type, *lasI* mutant, *rhlI* mutant, *pqsA* mutant, or *pqsH* mutant), *n* = 3. The value among each sample with different letters is significantly different (*p* < 0.05).

**FIGURE 2:**
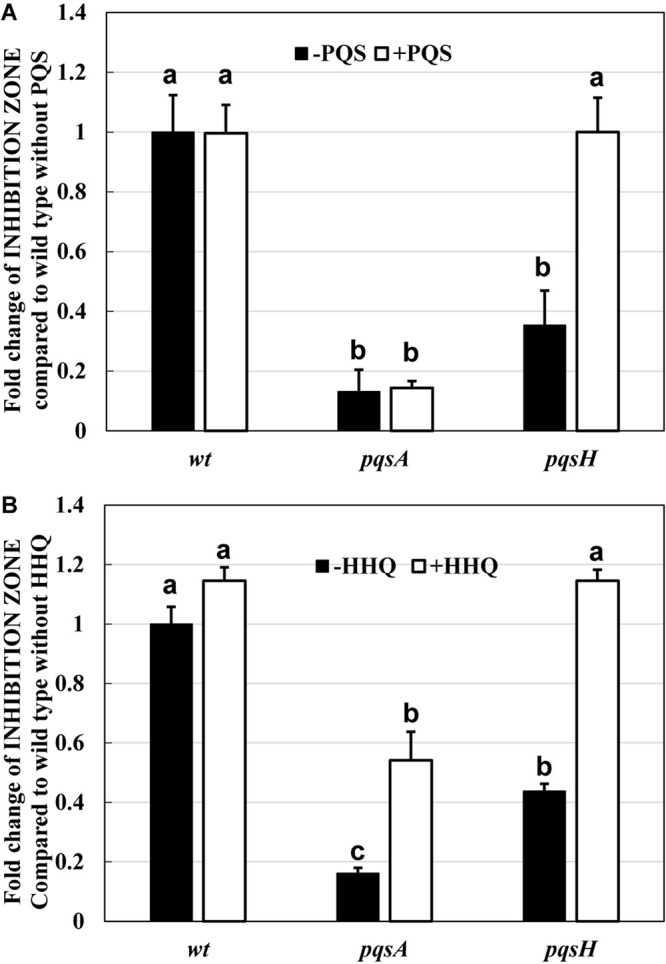
Restoration of the inhibitory effect by two quinolone compounds, PQS **(A)** and HHQ **(B)**, on the predation plate assays using a mixture of *Bdellovibrio bacteriovorus* 109J and *Escherichia coli* BW25113 in the presence of *Pseudomonas aeruginosa* wild type, *pqsA* mutant, or *pqsH* mutant. Each PQS or HHQ standard solution (solvent: DMSO, concentration: 160 nmol) or DMSO solution (control) was placed on the assay plate along with the cell culture of *P. aeruginosa* wild type, *pqsA* mutant, or *pqsH* mutant. All the plates were incubated at 30°C for 4 days. PQS, *Pseudomonas* quinolone signal, 2-heptyl-3-hydroxy-4(1H)-quinolone; HHQ, 2-heptyl-4-quinolone. *n* = 3. The value among each sample with different letters is significantly different (*p* < 0.05).

First, the effect of *P. aeruginosa* PA14 wild type on the predation of *E. coli* K-12 BW25113 by *B. bacteriovorus* was examined. Under regular conditions in the presence of *E. coli* and *B. bacteriovorus*, the number of *E. coli* cells decreased 1,000–10,000 times by the predation by *B. bacteriovorus* (Figure 1A). However, *B. bacteriovorus* could not prey *E. coli* cells in the presence of *P. aeruginosa* PA14 (Figure 1A). While the predatory activity of *B. bacteriovorus* was basically observed by checking a clear zone on the agar plate (Supplementary Figure 1A), the inhibition of predation by *P. aeruginosa* was seen on NB agar plates after incubation, as indicated in a result wherein *E. coli* cells grew in an area near to where the culture of *P. aeruginosa* PA14 was spotted on the agar plate (Supplementary Figure 1B). Without inoculating *B. bacteriovorus* cells, only the *E. coli* cells were grown on the agar plate (Supplementary Figure 1C): note that the reflection of the fluorescent lamp is more diffuse due to the turbidity of *E. coli* growth. Next, the involvement of *P*. *aeruginosa* QS in the predatory activity of *B. bacteriovorus* was investigated by using each QS mutant (*lasI*, *rhlI*, *pqsA*, or *pqsH*) since these genes are involved in the synthesis of each signal molecule. As shown in Figure 1B, whereas a large inhibitory zone was observed in the wild-type strain, *lasI* mutant, and *rhlI* mutant, the zone was reduced when *pqsA* or *pqsH* mutant was tested (Supplementary Figure 2, indicated in plate photos). The comparison clearly showed that the two mutants, *pqsA* and *pqsH*, do not have any inhibitory effect on the predation activity of *E. coli* by *B. bacteriovorus* (Figure 1B), whereas *lasI* and *rhlI* mutants showed almost the same predation inhibitory activity as the wild-type strain, PA14. Next, when the effect of a complementation assay with QS signal molecules, PQS and HHQ, on the predatory activity by *B. bacteriovorus* was investigated, the addition of PQS at the *pqsH* mutant or HHQ at the *pqsA* and *pqsH* mutants enhanced the inhibitory effect on the predation activity of *B. bacteriovorus* (Figure 2). In this experiment, the *pqsA* mutant strain was used as the strain that does not produce both PQS and HHQ, and the *pqsH* mutant strain was used as the strain that produces HHQ but not PQS. The recovery of predatory inhibitory activity in the *pqsA* and *pqsH* mutants was revealed by the size of the inhibition zone in the plate assays in the presence of PQS or HHQ (Supplementary Figure 3). Therefore, it was discovered that among the QS systems of *P. aeruginosa*, the *pqs* regulation system plays an essential role in inhibiting the predatory activity of *B. bacteriovorus*. HHQ rather than PQS is also more important to inhibit the predation activity of *B. bacteriovorus* because the inhibitory effect on the plate assays in the presence of HHQ was greater than that of PQS.

### Quinolone Signals Are Essential to the Predation Inhibitory Effect of *B. bacteriovorus*

**FIGURE 3:**
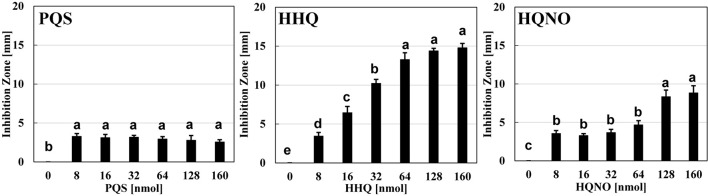
Effect of each quinolone compound itself on the predation plate assays using a mixture of *Bdellovibrio bacteriovorus* 109J and *Escherichia coli* BW25113. The inhibition zone for the predation plate assay using a mixture of *B. bacteriovorus* 109J and *E. coli* BW25113 was compared in the presence of PQS, HHQ, or HQNO compound (0, 8, 16, 32, 64, 128, or 160 nmol) placed on the plate after incubation at 30°C for 4 days. The 0-nmol sample is the control with DMSO alone. PQS, *Pseudomonas* quinolone signal, 2-heptyl-3-hydroxy-4(1H)-quinolone; HHQ, 2-heptyl-4-quinolone; HQNO, 2-heptyl-4-quinolinol 1-oxide. *n* = 3. The value among each sample with different letters is significantly different (*p* < 0.05).

**FIGURE 4:**
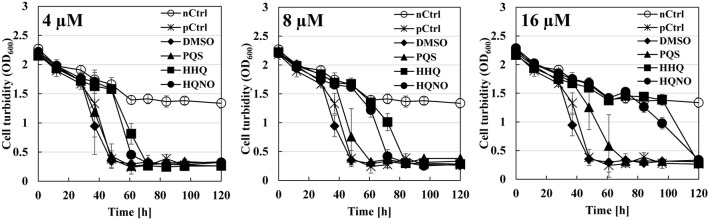
Effect of each quinolone compound itself on the predation liquid assays using a mixture of *Bdellovibrio bacteriovorus* 109J and *Escherichia coli* BW25113. Each cell turbidity of *E. coli* BW25113 (OD_600__*nm*_ ∼ 2) was monitored with time after inoculating the cells of *B. bacteriovorus* under 4 μM **(left)**, 8 μM **(middle)**, or 16 μM **(right)** of each PQS/HHQ/HQNO compound or DMSO which was used as a solvent for the compounds (as the control). “nCtrl” means a sample consisting of only the *E. coli* BW25113 cells in HEPES buffer without any compound, whereas “pCtrl” means a mixture of *E. coli* cells and *B. bacteriovorus* cells in HEPES buffer without any compound. PQS, *Pseudomonas* quinolone signal, 2-heptyl-3-hydroxy-4(1H)-quinolone; HHQ, 2-heptyl-4-quinolone; HQNO, 2-heptyl-4-quinolinol 1-oxide. *n* = 3.

The next question is whether the predation inhibitory activity to *B. bacteriovorus* is due to each signal molecule (PQS, HHQ, or HQNO) itself or the activation of the *pqs* regulation system by the exogenous addition of PQS, HHQ, or HQNO. First, the predatory activity of *B. bacteriovorus* was evaluated at a final amount of 8, 16, 32, 64, 128, and 160 nmol potential inhibitor by plate assays under the condition that only each *pqs* QS signal molecule (PQS, HHQ, or HQNO) was present without using *P. aeruginosa* culture. As a result, HHQ was the most effective compound in inhibiting the predatory activity of *B. bacteriovorus* (Figure 3 and Supplementary Figure 4). The inhibitory effect was even higher when tested at a final amount of 8, 16, 32, 64, 128, and 160 nmol. Moreover, 100% DMSO addition as the solvent control has no inhibition (Supplementary Figure 4). Additionally, the inhibitory effect of the PQS, HHQ, or HQNO was also evaluated at 4, 8, and 16 μM in assays using a liquid culture, by which the predation activity of *B. bacteriovorus* can be evaluated by reducing the turbidity mainly derived from *E. coli* cells. As shown in Figure 4, the predatory activity of *B. bacteriovorus* was inhibited according to the concentration of PQS, HHQ, or HQNO tested. The concentrations tested should be reasonable because a similar concentration of PQS, HHQ, and HQNO was detected from the culture of *P. aeruginosa* (Lesic et al., 2007). Particularly, HHQ and HQNO inhibited the activity of *B. bacteriovorus* compared to the control with DMSO. At higher concentrations (20, 40, 80, and 160 μM) of each quinolone compound, the inhibitory effect was also more clearly observed (Supplementary Figure 5), as shown in the result wherein complete inhibition of predation activity was found at high concentrations of HQNO (over 20 μM) and HHQ (over 40 μM). Moreover, since pyocyanin is known as a compound produced by the *pqs* QS system (Gallagher et al., 2002), the effect of pyocyanin on the predation activity was examined. Consequently, pyocyanin had no effect on the predation activity of *B. bacteriovorus* (Supplementary Figure 6). The results altogether indicate that PQS, HHQ, and HQNO produced by *P. aeruginosa* are essential compounds that inhibit the predation activity of *B. bacteriovorus* directly.

### Effect of Quinolone Compounds on *B. bacteriovorus* Cells

**FIGURE 5:**
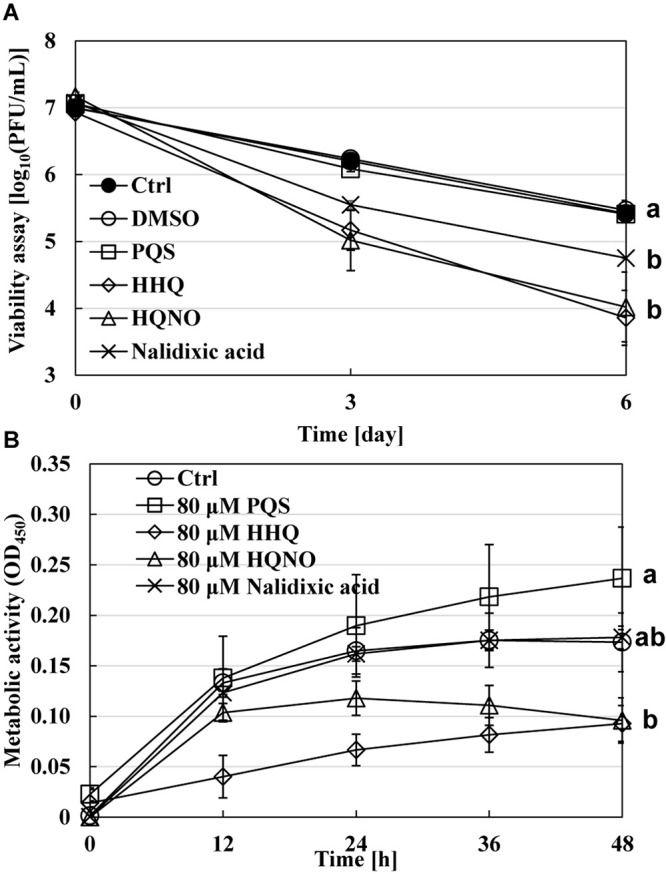
Effect of each quinolone compound itself on the viability of *Bdellovibrio bacteriovorus* cells. **(A)** The dynamics of viable cells of *B. bacteriovorus* in HEPES buffer with or without 80 μM of PQS/HHQ/HQNO/nalidixic acid. The incubation was conducted for 6 days at 30°C and 120 rpm under aerobic condition. *n* = 2. **(B)** The metabolic activity of *B. bacteriovarus* cells with or without 80 μM of PQS, HHQ, HQNO, or nalidixic acid for 48 h at 30°C left to stand in LB medium. “Ctrl” means a mixture of *B. bacteriovorus* cells and DMSO as a control solvent. PQS, *Pseudomonas* quinolone signal, 2-heptyl-3-hydroxy-4(1H)-quinolone; HHQ, 2-heptyl-4-quinolone; HQNO, 2-heptyl-4-quinolinol 1-oxide. *n* = 3. The value among each sample with different letters is significantly different (*p* < 0.05).

The next point is how quinolone compounds act directly on *B. bacteriovorus* cells in the absence of prey *E. coli* cells. The viability of *B. bacteriovorus* cells in HEPES buffer was monitored for 6 days with or without the addition of 80 μM of each quinolone compound, including nalidixic acid, one of the quinolone compounds, as a positive control. As a result, the number of *B. bacteriovorus* cells drastically reduced in the presence of HHQ or HQNO (Figure 5A). These compounds may affect the electron mediators of *B. bacteriovorus* since HQNO kills cells by flow inhibition of electrons, such as cytochrome *bc*_1_ complex (Hazan et al., 2016). Corroborating evidence was obtained from the results using the WST-8 colorimetric assay kit to evaluate the metabolic activity of *B. bacteriovorus* under HEPES buffer with or without 80 μM of each quinolone signal. As shown in Figure 5B, the metabolic activity of *B. bacteriovorus* was also reduced in the presence of HHQ or HQNO. Hence, our results indicate that quinolone signal compounds produced by *P. aeruginosa* act directly to inactivate *B. bacteriovorus* cells.

Additionally, it was reported that indole represses the predatory activity of *B. bacteriovorus* (Dwidar et al., 2015); it was tested whether *E. coli* can produce indole from several quinolone compounds derived from *P. aeruginosa*. Supplementary Figure 7 shows the indole production in *E. coli* in the presence of PQS, HHQ, or HQNO. As a result, there was no difference in the amount of indole produced with or without each quinolone compound. Therefore, it is fairly unlikely that the inhibition of the predatory activity of *B. bacteriovorus* may be caused by indole produced from quinolone compounds.

### Relationship Between *pqs* Regulation System and Predation Activity of *B. bacteriovorus*

**FIGURE 6:**
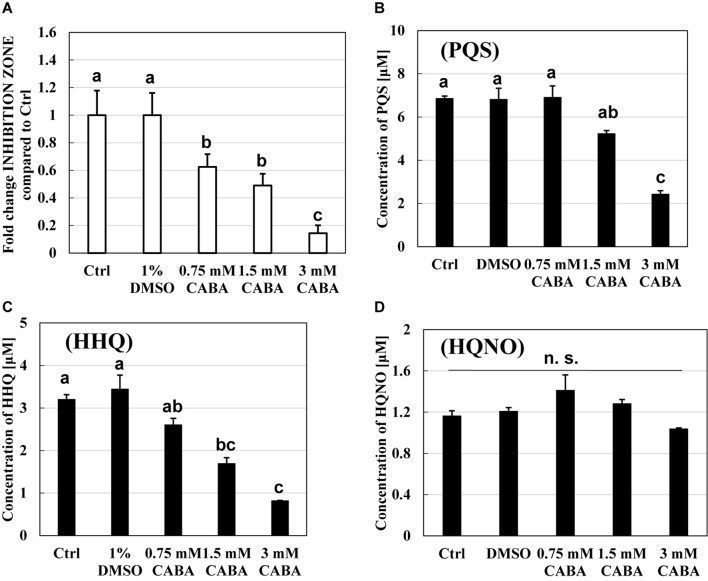
Effect of a *pqs* quorum-sensing inhibitor, 2-amino-6-chlorobenzoic acid (CABA), on the predation inhibition by *Pseudomonas aeruginosa* and the production of PQS, HHQ, or HQNO. **(A)** The reduction of predation inhibitory effect in the presence of CABA. The predation plate assays using a mixture of *Bdellovibrio bacteriovorus* 109J and *Escherichia coli* BW25113 were conducted at 30°C for 3 days in the presence of quinolone compounds extracted from the culture of *P. aeruginosa* wild type containing 0, 0.75, 1.5, or 3 mM of CABA. **(B)** PQS, **(C)** HHQ, and **(D)** HQNO concentration in the extracts of bacterial culture of *P. aeruginosa* PA14 after incubation at 37°C and 120 rpm for 10 h under LB medium with 0, 0.75, 1.5, or 3 mM of CABA. The concentration of HHQ extracted was determined by LC-QTOF-MS. Ctrl and 1% DMSO mean that each sample from the culture uses only LB medium or uses LB with 1% DMSO as solvent control. PQS, *Pseudomonas* quinolone signal, 2-heptyl-3-hydroxy-4(1H)-quinolone; HHQ, 2-heptyl-4-quinolone; HQNO, 2-heptyl-4-quinolinol 1-oxide. *n* = 3. The value among each sample with different letters is significantly different (*p* < 0.05). *n* = 3. n. s., not significant.

The relationship between the *pqs* regulation system and the predation activity of *B. bacteriovorus* was further examined using CABA, a *pqs* QS inhibitor (Lesic et al., 2007). Figure 6A shows the inhibitory effect of *B. bacteriovorus* at several concentrations (0.75, 1.5, and 3 mM CABA), at which the growth of *P. aeruginosa* was not inhibited (Supplementary Figure 8). The results clearly show that the predatory activity of *B. bacteriovorus* was not inhibited according to the increasing concentration of CABA. Additionally, the actual concentration of HHQ in each culture was verified by LC-QTOF-MS analyses (Figure 6C). As a result, lower concentrations of HHQ were detected from the cultures at higher concentrations of CABA. A similar tendency was observed for the PQS concentration but not for the HQNO one (Figures 6B,D). Because the concentration of HHQ is highly correlated with that of CABA compared to PQS and HQNO, the HHQ produced is a key factor to inhibit the predation activity of *B. bacteriovorus*.

### Effect of Quinolone Compounds on Other *B. bacteriovorus* Isolates

**FIGURE 7:**
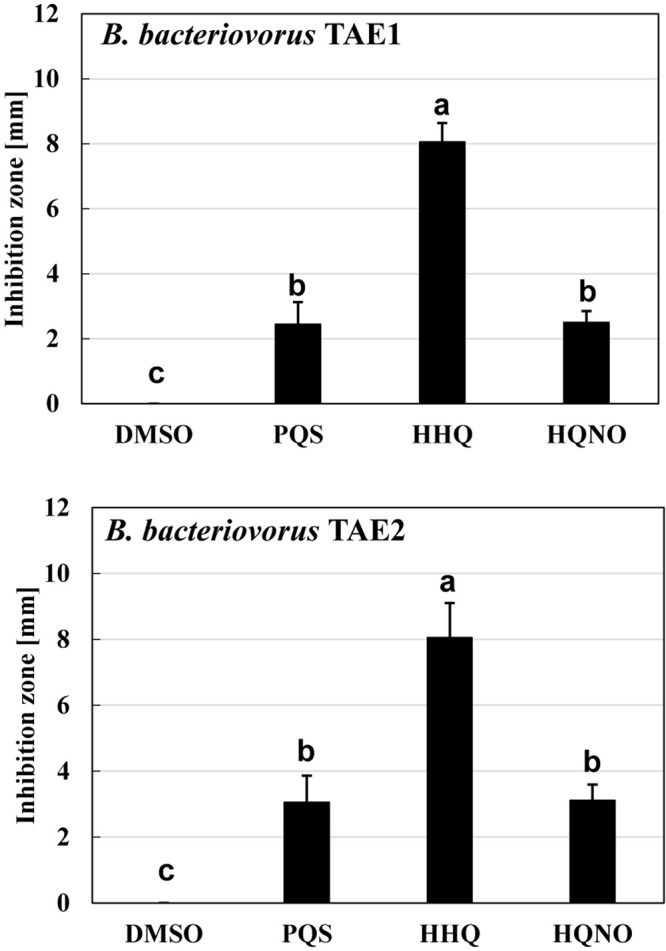
The effect of quinolone compounds on two environmental isolates of *Bdellovibrio bacteriovorus*. The predation plate assay was conducted using two isolates (TAE1 and TAE2) of *B. bacteriovorus*. The inhibition zone for the predation plate assay uses a mixture of each *B. bacteriovorus* isolate, and *Escherichia coli* BW25113 was compared in the presence of PQS, HHQ, or HQNO compound (16 nmol) placed on the plate after incubation at 30°C for 4 days. The DMSO sample is the solvent control with DMSO alone. PQS, *Pseudomonas* quinolone signal, 2-heptyl-3-hydroxy-4(1H)-quinolone; HHQ, 2-heptyl-4-quinolone; HQNO, 2-heptyl-4-quinolinol 1-oxide. *n* = 3. The value among each sample with different letters is significantly different (*p* < 0.05).

Another question is whether the inhibitory effect of *B. bacteriovorus* by the quinolone compounds occurs only in *B. bacteriovorus* strain 109J that has been used in this study. To answer the question, two strains of *Bdellovibrio-*like bacteria were isolated from a river sample and similarly evaluated for their effect on quinolone compounds. First, 16S ribosomal RNA gene homology analysis was conducted on these two strains, and it was discovered that both strains had 99% homology (TAE1: 99.36%, TAE2: 99.79%) with the *B. bacteriovorus* strain 109J complete sequence. Second, the two *B. bacteriovorus* strains were also inhibited in the presence of quinolone compounds (Figure 7). No inhibition zone is observed in the control condition with DMSO alone, as shown in Supplementary Figure 4. In both strains, HHQ showed the highest predation inhibitory effect. These results support the above-mentioned point that HHQ should be a key compound to inhibit the predation activity of *B. bacteriovorus*.

### Effect of *P. aeruginosa* Mutant Strains That Do Not Produce Quinolone Compounds on the Predation by *B. bacteriovorus*

Considering the previous results, it might be thought that *B. bacteriovorus* can prey on *P. aeruginosa* mutant strains that do not produce quinolone compounds. However, *P. aeruginosa* (*pqsA* or *pqsH* mutant) with a small inhibition zone in Figure 1B was not preyed upon by *B. bacteriovorus* when *P. aeruginosa* cell suspension was added to *B. bacteriovorus* (Supplementary Figure 9). For *P. aeruginosa* to be preyed upon by *B. bacteriovorus*, it was considered that not only quinolone compounds but also other predation inhibitors had to be avoided.

Next, it was investigated whether it would be possible for *B. bacteriovorus* to prey upon *E. coli* in the presence of *P. aeruginosa* mutant strains, which do not produce quinolone compounds. As a result, *B. bacteriovorus* was unable to prey upon *E. coli* even in the presence of *P. aeruginosa* mutant strains (*pqsA* or *pqsH*) with a small inhibition zone, as shown in Figure 1B (Supplementary Figure 10).

These results suggest that quinolone compounds are not the only factor inhibiting the predatory activity of *B. bacteriovorus* but that there are other factors.

## Discussion

The reason for the presence or absence of predatory activity against *P. aeruginosa* by *B. bacteriovorus* has still been a big mystery to date. *P. aeruginosa*, a Gram-negative bacterium like *E. coli*, has been reported to be preyed upon by *B. bacteriovorus* (Stolp and Starr, 1963; Shanks et al., 2013; Iebba et al., 2014; Pérez et al., 2016), while other researchers have reported such to be not preyed upon (Mukherjee et al., 2015). Since the different behavior regarding predatory activity was considered to be because of the barrier caused by *P. aeruginosa* rather than the property of *B. bacteriovorus* itself, we first investigated the relationship between each QS system derived from *P. aeruginosa* and the predatory activity of *B. bacteirovorus* in this study to narrow down the virulence factors produced by *P. aeruginosa* (Winson et al., 1995; Latifi et al., 1996; Pesci et al., 1997, 1999; McKnight et al., 2000; Gallagher et al., 2002; Kiratisin et al., 2002; Déziel et al., 2004; Xiao et al., 2006; Lee et al., 2013) against *B. bacteriovorus*. These results clearly show that quinolone compounds derived from *P. aeruginosa* negatively affect the predation of *E. coli* by *B. bacteriovorus* (Figures 1, 2, 3). For the complementary experiments shown in Figure 2, the *pqsA* mutant strain, which lacked the first step in the PQS synthesis pathway (Gallagher et al., 2002; Coleman et al., 2008), was not complemented by exogenous PQS since it could not produce HHQ, but the *pqsH* mutant strain, which lacked the conversion gene of HHQ to PQS at the last step in the PQS synthesis pathway (Gallagher et al., 2002; Déziel et al., 2004; Dubern and Diggle, 2008; Schertzer et al., 2009), was complemented by exogenous PQS since it could upregulate PQS synthesis (Xiao et al., 2006) and produce HHQ. However, it is considered to have been complemented in the *pqsH* mutant strain with exogenous HHQ since *pqsA* is upregulated by PqsR receiving HHQ (Rampioni et al., 2016). However, for the *pqsA* mutant strain, the size of the inhibition zone may be due to the exogenous HHQ itself, as shown in Figure 3, rather than complementation. Additionally, among the quinolone compounds, HHQ and HQNO directly inactivated the activity of *B. bacteriovorus* (Figures 4, 5). In general, similar phenomena likely occur in nature since *P. aeruginosa* produces 4–40 μM PQS, 0.5–4 μM HHQ, and ∼75 μM HQNO in a pure culture system (Lépine et al., 2004; Lesic et al., 2007; Ji et al., 2016). As the inhibitory mechanism, it inhibits the activity of electron mediators such as cytochrome *bc*_1_ complex (Hazan et al., 2016). However, HHQ exhibits bacteriostatic activity in a species-specific manner (Reen et al., 2011), but the detailed mechanism is still unknown. Figure 4 and Supplementary Figure 6 suggest that HHQ (over 40 μM) and HQNO (over 20 μM) are inhibited, but HHQ is as effective as or more effective than HQNO at lower concentrations (4–16 μM). Similarly, in the plate experiment (Figure 3), only HHQ showed a more significant inhibition zone than PQS at a low number of compounds (16–32 nmol), while not only HHQ but also HQNO showed a larger inhibition zone than PQS at high amounts of compounds (over 64 nmol). Considering the solubility of each compound in liquid conditions and its diffusion on the plate, the inhibitory mechanism of each quinolone compound against *B. bacteriovorus* needs to be clarified in the future. The effects of HHQ and HQNO are almost the same; however, the concentration of HHQ produced is higher than that of HQNO in the actual culture of *P. aeruginosa* (Figure 6A). Additionally, the predatory activity of *B. bacteriovorus* was restored in the presence of a *pqs* regulation inhibitor (Lesic et al., 2007), CABA (Figures 6B,D). Thus, the inhibition of predatory activity by HHQ was clarified as a barrier mechanism of *P. aeruginosa* against *B. bacteriovorus*. Since HHQ, which had the highest inhibition in the plate assay, was the focus of this study, instrumental analysis was conducted using the *P. aeruginosa* culture after about 10 h. Therefore, it looks like only the HHQ concentration correlated with the size of the inhibition zone. This may also correlate with the degree of inhibition of HQNO when *P. aeruginosa* is incubated for a longer time (data not shown). Since these *pqs* QS signals produced by *P. aeruginosa* are known to act as not only the regulation of gene expression but also an antibiotic for some bacterial species (Reen et al., 2011; Szamosvári et al., 2019), the predation activity assay of *B. bacteriovorus* strains tested may be inactivated as shown in Figure 7.

Although the *pqs* regulation system of *P. aeruginosa* is one of the defense mechanisms against *B. bacteriovorus*, this predator was unable to prey on *pqs* mutants that could not produce quinolone compounds such as HHQ (Supplementary Figure 9). The results show that *P. aeruginosa* may have another mechanism that evades the attack of *B. bacteriovorus*. In the predation evaluation by plate assays, the inhibition zone of *pqs* mutants was small, suggesting a different inhibitory mechanism other than extracellular secretory substances. Further research is needed to elucidate the mechanism of the presence or absence of predatory activity of *B. bacteriovorus* against *P. aeruginosa*.

So far, it has been reported that indole, which is one of the QS molecules, and cyanide inhibits the activity of *B. bacteriovorus* (Dwidar et al., 2015; Mun et al., 2017); however, there is no knowledge about its relationship with other QS compounds. Other than that, Varon and Shilo (1981) reported that the predation of *B. bacteriovorus* was inhibited by sodium dodecyl sulfate (SDS). Surfactants, not limited to artificially added SDS, are produced by various microorganisms such as bacteria and yeast. In Supplementary Figures 9, 10, in the presence of the quinolone compound non-producing *P. aeruginosa* strain, *B. bacteriovorus* could not prey upon not only *P. aeruginosa* but also *E. coli*. Thus, the strategy should not be to avoid only one of the extracellular inhibitors to allow *B. bacteriovorus* to prey upon *P. aeruginosa*. As a summary of this research, our results showed that quinolone signals related to the QS of *P. aeruginosa*, especially HHQ and HQNO, inhibit the predation activity of *B. bacteriovorus*. Among the quinolone compounds, HHQ was the most effective quinolone compound against *B. bacteriovorus*. It was discovered that quinolone compounds such as HHQ act as bactericidal on *B. bacteriovorus*, unlike indole that suppresses viability without killing it. As shown in the results of this research, the accumulation of knowledge to understand the relationship between predator bacteria and other bacteria is indispensable for the application and usage of *B. bacteriovorus* in the future.

## Data Availability Statement

The raw data supporting the conclusions of this article will be made available by the authors, without undue reservation.

## Author Contributions

YH, YN, TMo, and KK performed the experiments. TMa, KK, and RG-C supervised the project. YH initially wrote the manuscript. TMa revised the manuscript. All authors designed the study, discussed the results, contributed to the article, and approved the submitted version.

## Conflict of Interest

The authors declare that the research was conducted in the absence of any commercial or financial relationships that could be construed as a potential conflict of interest.

## Publisher’s Note

All claims expressed in this article are solely those of the authors and do not necessarily represent those of their affiliated organizations, or those of the publisher, the editors and the reviewers. Any product that may be evaluated in this article, or claim that may be made by its manufacturer, is not guaranteed or endorsed by the publisher.

## References

[B1] Baba T. Ara T. Hasegawa M. Takai Y. Okumura Y. Baba M. (2006). Construction of *Escherichia coli* K-12 in-frame, single-gene knockout mutants: the Keio collection. *Mol. Syst. Biol.* 2. 10.1038/msb4100050 16738554 PMC1681482

[B2] Burgess J. G. Jordan E. M. Bregu M. Mearns-Spragg A. Boyd K. G. (1999). Microbial antagonism: a neglected avenue of natural products research. *J. Biotechnol.* 70 27–32. 10412203 10.1016/s0168-1656(99)00054-1

[B3] Chen H. Brinkac L. M. Mishra P. Li N. Lymperopoulou D. S. Dickerson T. L. (2015). Draft genome sequences for the obligate bacterial predators *Bacteriovorax* spp. of four phylogenetic clusters. *Stand. Genomic Sci.* 10:11. 10.1186/1944-3277-10-11 26203326 PMC4511183

[B4] Coleman J. P. Hudson L. L. McKnight S. L. Farrow J. M. Calfee M. W. Lindsey C. A. (2008). *Pseudomonas aeruginosa* PqsA is an anthranilate-coenzyme A ligase. *J. Bacteriol.* 190 1247–1255. 10.1128/JB.01140-07 18083812 PMC2238192

[B5] Dashiff A. Junka R. A. Libera M. Kadouri D. E. (2011). Predation of human pathogens by the predatory bacteria *Micavibrio aeruginosavorus* and *Bdellovibrio bacteriovorus*. *J. Appl. Microbiol.* 110 431–444. 10.1111/j.1365-2672.2010.04900.x 21114596

[B6] Datsenko K. A. Wanner B. L. (2000). One-step inactivation of chromosomal genes in *Escherichia coli* K-12 using PCR products. *Proc. Natl. Acad. Sci. U.S.A.* 97 6640–6645. 10.1073/pnas.120163297 10829079 PMC18686

[B7] de Almeida F. A. Carneiro D. G. de Oliveira Mendes T. A. Barros E. Pinto U. M. de Oliveira L. L. (2018). N-dodecanoyl-homoserine lactone influences the levels of thiol and proteins related to oxidation-reduction process in *Salmonella*. *PLoS One* 13:e0204673. 10.1371/journal.pone.0204673 30304064 PMC6179229

[B8] Déziel E. Lépine F. Milot S. He J. Mindrinos M. N. Tompkins R. G. (2004). Analysis of *Pseudomonas aeruginosa* 4-hydroxy-2-alkylquinolines (HAQs) reveals a role for 4-hydroxy-2-heptylquinoline in cell-to-cell communication. *Proc. Natl. Acad. Sci. U.S.A.* 101 1339–1344. 10.1073/pnas.0307694100 14739337 PMC337054

[B9] Dubern J. F. Diggle S. P. (2008). Quorum sensing by 2-alkyl-4-quinolones in *Pseudomonas aeruginosa* and other bacterial species. *Mol. Biosyst.* 4 882–888. 10.1039/b803796p 18704225

[B10] Dubuis C. Keel C. Haas D. (2007). Dialogues of root-colonizing biocontrol peudomonads. *Eur. J. Plant Pathol.* 119 311–328. 10.1007/s10658-007-9157-1

[B11] Dwidar M. Nam D. Mitchell R. J. (2015). Indole negatively impacts predation by *Bdellovibrio bacteriovorus* and its release from the bdelloplast. *Environ. Microbiol.* 17 1009–1022. 10.1111/1462-2920.12463 24673893

[B12] Essar D. W. Eberly L. Hadero A. Crawford I. P. (1990). Identification and characterization of genes for a second anthranilate synthase in *Pseudomonas aeruginosa*: interchangeability of the two anthranilate synthase and evolutionary implications. *J. Bacteriol.* 172 884–900. 10.1128/jb.172.2.884-900.1990 2153661 PMC208517

[B13] Evans K. J. Lambert C. Sockett R. E. (2007). Predation by *Bdellovibrio bacteriovorus* HD100 requires type IV pili. *J. Bacteriol.* 189 4850–4859. 10.1128/JB.01942-06 17416646 PMC1913455

[B14] Feng S. Tan C. H. Cohen Y. Rice S. A. (2016). Isolation of *Bdellovibrio bacteriovorus* from a tropical wastewater treatment plant and predation of mixed species biofilms assembled by the native community members. *Environ. Microbiol.* 18 3923–3931. 10.1111/1462-2920.13384 27328268

[B15] Fratamico P. M. Cooke P. H. (1996). Isolation of *Bdellovibrios* that prey on *Escherichia coli* O157:H7 and *Salmonella* species and application for removal of prey from stainless steel surfaces. *J. Food Saf.* 16 161–173. 10.1111/j.1745-4565.1996.tb00157.x

[B16] Fuqua C. Parsek M. R. Greenberg E. P. (2001). Regulation of gene expression by cell-to-cell communication: acyl-homoserine lactone quorum sensing. *Annu. Rev. Genet.* 35 439–468. 10.1146/annurev.genet.35.102401.090913 11700290

[B17] Fuqua W. C. Winans S. C. Greenberg E. P. (1994). Quorum sensing in bacteria: the LuxR-LuxI family of cell density- responsive transcriptional regulators. *J. Bacteriol.* 176 269–275. 10.1128/jb.176.2.269-275.1994 8288518 PMC205046

[B18] Gallagher L. A. McKnight S. L. Kuznetsova M. S. Pesci E. C. Manoil C. (2002). Functions required for extracellular quinolone signaling by *Pseudomonas aeruginosa*. *J. Bacteriol.* 184 6472–6480. 10.1128/JB.184.23.6472-6480.2002 12426334 PMC135424

[B19] Galloway W. R. J. D. Hodgkinson J. T. Bowden S. D. Welch M. Spring D. R. (2011). Quorum sensing in Gram-negative bacteria: small-molecule modulation of AHL and AI-2 quorum sensing pathways. *Chem. Rev.* 111 28–67. 10.1021/cr100109t 21182299

[B20] Hazan R. Que Y. A. Maura D. Strobel B. Majcherczyk P. A. Hopper L. R. (2016). Auto poisoning of the respiratory chain by a quorum-sensing-regulated molecule favors biofilm formation and antibiotic tolerance. *Curr. Biol.* 26 195–206. 10.1016/j.cub.2015.11.056 26776731 PMC4729643

[B21] Hobley L. Lerner T. R. Williams L. E. Lambert C. Till R. Milner D. S. (2012). Genome analysis of a simultaneously predatory and prey-independent, novel *Bdellovibrio bacteriovorus* from the River Tiber, supports in silico predictions of both ancient and recent lateral gene transfer from diverse bacteria. *BMC Genomics* 13:670. 10.1186/1471-2164-13-670 23181807 PMC3539863

[B22] Huang J. Shi Y. Zeng G. Gu Y. Chen G. Shi L. (2016). Acyl-homoserine lactone-based quorum sensing and quorum quenching hold promise to determine the performance of biological wastewater treatments: an overview. *Chemosphere* 157 137–151. 10.1016/j.chemosphere.2016.05.032 27213243

[B23] Iebba V. Totino V. Santangelo F. Gagliardi A. Ciotoli L. Virga A. (2014). *Bdellovibrio bacteriovorus* directly attacks *Pseudomonas aeruginosa* and Staphylococcus aureus cystic fibrosis isolates. *Front. Microbiol.* 5:280. 10.3389/fmicb.2014.00280 24926292 PMC4046265

[B24] Im H. Dwidar M. Mitchell R. J. (2018). *Bdellovibrio bacteriovorus* HD100, a predator of Gram-negative bacteria, benefits energetically from *Staphylococcus aureus* biofilms without predation. *ISME J.* 12 2090–2095. 10.1038/s41396-018-0154-5 29849167 PMC6052163

[B25] Ji C. Sharma I. Pratihar D. Hudson L. L. Maura D. Guney T. (2016). Designed small-molecule inhibitors of the anthranilyl-CoA synthetase PqsA block quinolone biosynthesis in *Pseudomonas aeruginosa*. *ACS Chem. Biol.* 11 3061–3067. 10.1021/acschembio.6b00575 27658001 PMC5117135

[B26] Johansen P. Jespersen L. (2017). Impact of quorum sensing on the quality of fermented foods. *Curr. Opin. Food Sci.* 13 16–25. 10.1016/j.cofs.2017.01.001

[B27] Jurkevitch E. Minz D. Ramati B. Barel G. (2000). Prey range characterization, ribotyping, and diversity of soil and rhizosphere *Bdellovibrio* spp. isolated on phytopathogenic bacteria. *Appl. Environ. Microbiol.* 66 2365–2371. 10.1128/AEM.66.6.2365-2371.2000 10831412 PMC110534

[B28] Kadokami K. Ueno D. (2019). Comprehensive Target Analysis for 484 Organic Micropollutants in Environmental Waters by the Combination of Tandem Solid-Phase Extraction and Quadrupole Time-of-Flight Mass Spectrometry with Sequential Window Acquisition of All Theoretical Fragment-Ion Spectra Acquisition. *Anal. Chem.* 91 7749–7755. 10.1021/acs.analchem.9b01141 31132244

[B29] Kadouri D. O’Toole G. A. (2005). Susceptibility of biofilms to *Bdellovibrio bacteriovorus* attack. *Appl. Environ. Microbiol.* 71 4044–4051. 10.1128/AEM.71.7.4044-4051.2005 16000819 PMC1169041

[B30] Karlin D. A. Mastromarino A. J. Jones R. D. Stroehlein J. R. Lorentz O. (1985). Fecal skatole and indole and breath methane and hydrogen in patients with large bowel polyps or cancer. *J. Cancer Res. Clin. Oncol.* 109 135–141. 10.1007/BF00391888 3980562 PMC12253221

[B31] Kawamura-Sato K. Shibayama K. Horii T. Iimuma Y. Arakawa Y. Ohta M. (1999). Role of multiple efflux pumps in *Escherichia coli* in indole expulsion. *FEMS Microbiol. Lett.* 179 345–352. 10.1111/j.1574-6968.1999.tb08748.x 10518736

[B32] Kiratisin P. Tucker K. D. Passador L. (2002). LasR, a transcriptional activator of *Pseudomonas aeruginosa* virulence genes, functions as a multimer. *J. Bacteriol.* 184 4912–4919. 10.1128/JB.184.17.4912-4919.2002 12169617 PMC135272

[B33] Koval S. F. Hynes S. H. Flannagan R. S. Pasternak Z. Davidov Y. Jurkevitch E. (2013). *Bdellovibrio exovorus* sp. nov., a novel predator of *Caulobacter crescentus*. *Int. J. Syst. Evol. Microbiol.* 63 146–151. 10.1099/ijs.0.039701-0 22368169

[B34] Kuru E. Lambert C. Rittichier J. Till R. Ducret A. Derouaux A. (2017). Fluorescent D-amino-acids reveal bi-cellular cell wall modifications important for *Bdellovibrio bacteriovorus* predation. *Nat. Microbiol.* 2 1648–1657. 10.1038/s41564-017-0029-y 28974693 PMC5705579

[B35] Latifi A. Foglino M. Tanaka K. Williams P. Lazdunski A. (1996). A hierarchical quorum-sensing cascade in *Pseudomonas aeruginosa* links the transcriptional activators LasR and RhIR (VsmR) to expression of the stationary-phase sigma factor RpoS. *Mol. Microbiol.* 21 1137–1146. 10.1046/j.1365-2958.1996.00063.x 8898383

[B36] Lee J. Wu J. Deng Y. Wang J. Wang C. Wang J. (2013). A cell-cell communication signal integrates quorum sensing and stress response. *Nat. Chem. Biol.* 9 339–343. 10.1038/nchembio.1225 23542643

[B37] Lee J. H. Wood T. K. Lee J. (2015). Roles of indole as an interspecies and interkingdom signaling molecule. *Trends Microbiol.* 23 707–718. 10.1016/j.tim.2015.08.001 26439294

[B38] Lépine F. Milot S. Déziel E. He J. Rahme L. G. (2004). Electrospray/mass spectrometric identification and analysis of 4-hydroxy-2-alkylquinolines (HAQs) produced by *Pseudomonas aeruginosa*. *J. Am. Soc. Mass Spectrom.* 15 862–869. 10.1016/j.jasms.2004.02.012 15144975

[B39] Lerner T. R. Lovering A. L. Bui N. K. Uchida K. Aizawa S. I. Vollmer W. (2012). Specialized peptidoglycan hydrolases sculpt the intra-bacterial niche of predatory *Bdellovibrio* and increase population fitness. *PLoS Pathog.* 8:e1002524. 10.1371/journal.ppat.1002524 22346754 PMC3276566

[B40] Lesic B. Lépine F. Déziel E. Zhang J. Zhang Q. Padfield K. (2007). Inhibitors of pathogen intercellular signals as selective anti-infective compounds. *PLoS Pathog.* 3:e0030126. 10.1371/journal.ppat.0030126 17941706 PMC2323289

[B41] Liberati N. T. Urbach J. M. Miyata S. Lee D. G. Drenkard E. Wu G. (2006). An ordered, nonredundant library of *Pseudomonas aeruginosa* strain PA14 transposon insertion mutants. *Proc. Natl. Acad. Sci. U.S.A.* 103 2833–2838. 10.1073/pnas.0511100103 16477005 PMC1413827

[B42] Liu Y. C. Chan K. G. Chang C. Y. (2015). Modulation of host biology by *Pseudomonas aeruginosa* quorum sensing signal molecules: messengers or traitors. *Front. Microbiol.* 6:1226. 10.3389/fmicb.2015.01226 26617576 PMC4637427

[B43] Mahmoud K. K. Koval S. F. (2010). Characterization of type IV pili in the life cycle of the predator bacterium *Bdellovibrio*. *Microbiology* 156 1040–1051. 10.1099/mic.0.036137-0 20056705

[B44] McKnight S. L. Iglewski B. H. Pesci E. C. (2000). The *Pseudomonas* quinolone signal regulates rhl quorum sensing in *Pseudomonas aeruginosa*. *J. Bacteriol.* 182 2702–2708. 10.1128/JB.182.10.2702-2708.2000 10781536 PMC101972

[B45] Miller M. B. Bassler B. L. (2001). Quorum sensing in bacteria. *Annu. Rev. Microbiol.* 55 165–199. 10.1146/annurev.micro.55.1.165 11544353

[B46] Mukherjee S. Brothers K. M. Shanks R. M. Q. Kadouri D. E. (2015). Visualizing *Bdellovibrio bacteriovorus* by using the tdTomato fluorescent protein. *Appl. Environ. Microbiol.* 82 1653–1661. 10.1128/AEM.03611-15 26712556 PMC4784026

[B47] Mun W. Kwon H. Im H. Choi S. Y. Monnappa A. K. Mitchell R. J. (2017). Cyanide production by chromobacterium piscinae shields it from *Bdellovibrio* bacteriovorus HD100 predation. *MBio.* 10.1128/mBio.01370-17 29259082 PMC5736907

[B48] O’Malley Y. Q. Reszka K. J. Spitz D. R. Denning G. M. Britigan B. E. (2004). *Pseudomonas aeruginosa* pyocyanin directly oxidizes glutathione and decreases its levels in airway epithelial cells. *Am. J. Physiol. Lung Cell. Mol. Physiol.* 287 L94–L103. 15020296 10.1152/ajplung.00025.2004

[B49] Page J. A. Lubbers B. Maher J. Ritsch L. Gragg S. E. (2015). Investigation into the efficacy of *Bdellovibrio bacteriovorus* as a novel preharvest intervention to control *Escherichia coli* O157:H7 and *Salmonella* in cattle using an in vitro model. *J. Food Prot.* 78 1745–1749. 10.4315/0362-028X.JFP-15-016 26319730

[B50] Pérez J. Moraleda-Muñoz A. Marcos-Torres F. J. Muñoz-Dorado J. (2016). Bacterial predation: 75 years and counting! *Environ. Microbiol.* 18 766–779. 10.1111/1462-2920.13171 26663201

[B51] Pesci E. C. Milbank J. B. J. Pearson J. P. Mcknight S. Kende A. S. Greenberg E. P. (1999). Quinolone signaling in the cell-to-cell communication system of *Pseudomonas aeruginosa*. *Proc. Natl. Acad. Sci. U.S.A.* 96 11229–11234. 10.1073/pnas.96.20.11229 10500159 PMC18016

[B52] Pesci E. C. Pearson J. P. Seed P. C. Iglewski B. H. (1997). Regulation of las and rhl quorum sensing in *Pseudomonas aeruginosa*. *J. Bacteriol.* 179 3127–3132. 10.1128/jb.179.10.3127-3132.1997 9150205 PMC179088

[B53] Pineiro S. A. Stine O. C. Chauhan A. Steyert S. R. Smith R. Williams H. N. (2007). Global survey of diversity among environmental saltwater *Bacteriovoracaceae*. *Environ. Microbiol.* 9 2441–2450. 10.1111/j.1462-2920.2007.01362.x 17803770

[B54] Rampioni G. Falcone M. Heeb S. Frangipani E. Fletcher M. P. Dubern J. F. (2016). Unravelling the genome-wide contributions of Specific 2-Alkyl-4-Quinolones and PqsE to quorum sensing in *Pseudomonas aeruginosa*. *PLoS Pathog.* 12:e1006029. 10.1371/journal.ppat.1006029 27851827 PMC5112799

[B55] Reen F. J. Mooij M. J. Holcombe L. J. Mcsweeney C. M. Mcglacken G. P. Morrissey J. P. (2011). The *Pseudomonas* quinolone signal (PQS), and its precursor HHQ, modulate interspecies and interkingdom behaviour. *FEMS Microbiol. Ecol.* 77 413–428. 10.1111/j.1574-6941.2011.01121.x 21539583

[B56] Sambrook J. Fritsch E. F. Maniatis T. (1989). *Molecular Cloning: A Laboratory Manual.* Cold Spring Harbor, NY: Cold Spring Harbor Laboratory Press.

[B57] Schertzer J. W. Boulette M. L. Whiteley M. (2009). More than a signal: non-signaling properties of quorum sensing molecules. *Trends Microbiol.* 17 189–195. 10.1016/j.tim.2009.02.001 19375323

[B58] Semblante G. U. Phan H. V. Hai F. I. Xu Z. Q. Price W. E. Nghiem L. D. (2017). The role of microbial diversity and composition in minimizing sludge production in the oxic-settling-anoxic process. *Sci. Total Environ.* 60 558–567. 10.1016/j.scitotenv.2017.06.253 28704678

[B59] Shanks R. M. Q. Davra V. R. Romanowski E. G. Brothers K. M. Stella N. A. Godboley D. (2013). An eye to a kill: using predatory bacteria to control Gram-negative pathogens associated with ocular infections. *PLoS One* 8:e66723. 10.1371/journal.pone.0066723 23824756 PMC3688930

[B60] Shatzkes K. Chae R. Tang C. Ramirez G. C. Mukherjee S. Tsenova L. (2015). Examining the safety of respiratory and intravenous inoculation of *Bdellovibrio bacteriovorus* and *Micavibrio aeruginosavorus* in a mouse model. *Sci. Rep.* 5:12899. 10.1038/srep12899 26250699 PMC4528228

[B61] Sockett R. E. Lambert C. (2004). *Bdellovibrio* as therapeutic agents: A predatory renaissance? *Nat. Rev. Microbiol.* 2 669–675. 10.1038/nrmicro959 15263901

[B62] Stolp H. Starr M. P. (1963). *Bdellovibrio bacteriovorus* gen. et sp. n., a predatory, ectoparasitic, and bacteriolytic microorganism. Antonie Van Leeuwenhoek 29 217–248. 10.1007/BF02046064 14068454

[B63] Stolp H. Starr M. P. (1965). Bacteriolysis. *Annu. Rev. Microbiol.* 19 79–104. 5318449 10.1146/annurev.mi.19.100165.000455

[B64] Szamosvári D. Schuhmacher T. Hauck C. R. Böttcher T. (2019). A thiochromenone antibiotic derived from the *Pseudomonas* quinolone signal selectively targets the Gram-negative pathogen *Moraxella catarrhalis*. *Chem. Sci.* 10 6624–6628. 10.1039/c9sc01090d 31367314 PMC6624978

[B65] Tang B. L. Yang J. Chen X. L. Wang P. Zhao H. L. Su H. N. (2020). A predator–prey interaction between a marine *Pseudoalteromonas* sp. and Gram-positive bacteria. *Nat. Commun.* 11:285. 10.1038/s41467-019-14133-x 31941905 PMC6962226

[B66] Thomashow M. F. Rittenberg S. C. (1978a). Intraperiplasmic growth of *Bdellovibrio bacteriovorus* 109J: attachment of long-chain fatty acids to *Escherichia coli* peptidoglycan. *J. Bacteriol.* 135 1015–1023. 10.1128/jb.135.3.1015-1023.1978 357411 PMC222478

[B67] Thomashow M. F. Rittenberg S. C. (1978b). Intraperiplasmic growth of *Bdellovibrio bacteriovorus* 109J: solubilization of *Escherichia coli* peptidoglycan. *J. Bacteriol.* 135 998–1007. 10.1128/jb.135.3.998-1007.1978 357428 PMC222476

[B68] Varon M. Shilo M. (1981). Inhibition of the predatory activity of Bdellovibrio by various environmental pollutants. *Microbial. Ecology.* 10.1007/BF0203249 24227420

[B69] Wang D. Ding X. Rather P. N. (2001). Indole can act as an extracellular signal in *Escherichia coli*. *J. Bacteriol.* 183 4210–4216. 10.1128/JB.183.14.4210-4216.2001 11418561 PMC95310

[B70] Winson M. K. Camarat M. Latifi A. Foglino M. Chhabrat S. R. Daykin M. (1995). Multiple N-acyl-L-homoserine lactone signal molecules regulate production of virulence determinants and secondary metabolites in *Pseudomonas aeruginosa*. *Proc. Natl. Acad. Sci. U.S.A.* 92 9427–9431. 7568146 10.1073/pnas.92.20.9427 PMC40998

[B71] Withers H. Swift S. Williams P. (2001). Quorum sensing as an integral component of gene regulatory networks in gram-negative bacteria. *Curr. Opin. Microbiol.* 4 186–193. 10.1016/S1369-5274(00)00187-9 11282475

[B72] Xiao G. Déziel E. He J. Lépine F. Lesic B. Castonguay M. H. (2006). MvfR, a key *Pseudomonas aeruginosa* pathogenicity LTTR-class regulatory protein, has dual ligands. *Mol. Microbiol.* 62 1689–1699. 10.1111/j.1365-2958.2006.05462.x 17083468

[B73] Zuccato E. Venturi M. Di Leo G. Colombo L. Bertolo C. Doldi S. B. (1993). Role of bile acids and metabolic activity of colonic bacteria in increased risk of colon cancer after cholecystectomy. *Dig. Dis. Sci.* 38 514–519. 10.1007/BF01316508 8444084

