# Supplementary material for: Quinolone Signals Related to Pseudomonas Quinolone Signal-Quorum Sensing Inhibits the Predatory Activity of Bdellovibrio bacteriovorus
Source: Front Microbiol. 2021 Sep 10;12:722579. doi: 10.3389/fmicb.2021.722579 (PMC8461301; doi:10.3389/fmicb.2021.722579)
Supplement: Supplementary file 1 [file Data_Sheet_1.pdf]

Supplementary tables and figures

Table S1. LC-QTOF-MS conditions to check each concentration of quinolone compound in the fraction after the extraction from PA14 overnight culture with/without CABA, a *pqs* quorum sensing inhibitor.

**Instrument**		Sciex X500R QTOF system		
**Column**		GL Science InertSustain AQ-C18 HP (3 µm, 2.1 × 150 mm)		
**Colomn temp.**		40˚C		
**Flow rate**		0.3 mL/min		
**Mobile phase**		(A) 5 mM CH_3COONH_4 in H_2O<br>(B) 5 mM CH_3COONH_4 in MeOH		
**Gradient profile**	**Time [min]**	0	15-25	25.1-30
	**B [%]**	50	95	50
**Injection volume**		2 µL		
**Ion source**		TurboIonSpray		
**Ionization**		ESI-positive		
**Measurement mode**		Swath		
**TOF-MS (scan range) ***		50 - 1 000 Da, 0.1 sec		
**TOF MS/MS**		50 - 1 000 Da, 22 ranges, 0.07 sec each		
**Collision energy ramp**		20-50 V		
**Mass resolution**		30 000		
**Total cycle time**		1.768 sec		

*The TOF-MS scan range was divided by 22 ranges. SWATH window indicates **Table S2**.

Table S2. SWATH windows to detect each quinolone compound by using LC-QTOF-MS.

No.	Window [Da]
**1**	50 - 150.5
**2**	149.5 - 213.7
**3**	212.7 - 230.1
**4**	229.1 - 245.8
**5**	244.8 - 269.1
**6**	268.1 - 289.1
**7**	288.1 - 304.7
**8**	303.7 - 316
**9**	315 - 326.9
**10**	325.9 - 338.2
**11**	337.2 - 349.1
**12**	348.1 - 361.5
**13**	360.5 - 375
**14**	374 - 387.7
**15**	386.7 - 400.5
**16**	399.5 - 412.1
**17**	411.1 - 424.9
**18**	423.9 - 440.5
**19**	439.5 - 474.7
**20**	473.7 - 509.3
**21**	508.3 - 891.5
**22**	890.5 - 1 000

Table S3. The ion transitions of each quinolone compound for the identification by using TOF MS and TOF MS/MS. PQS; Pseudomonas quinolone signal, 2-Heptyl-3-hydroxy-4(1H)-quinolone. HHQ; 2-Heptyl-4-quinolone. HQNO; 2-heptyl-4-quinolinol 1-oxide.

Compounds	Precursor ions	Product ions
**PQS**	260.164	188.070, 175.063, 147.067
**HHQ**	244.170	172.076, 159.068
**HQNO**	260.165	186.091, 159.068, 144.045

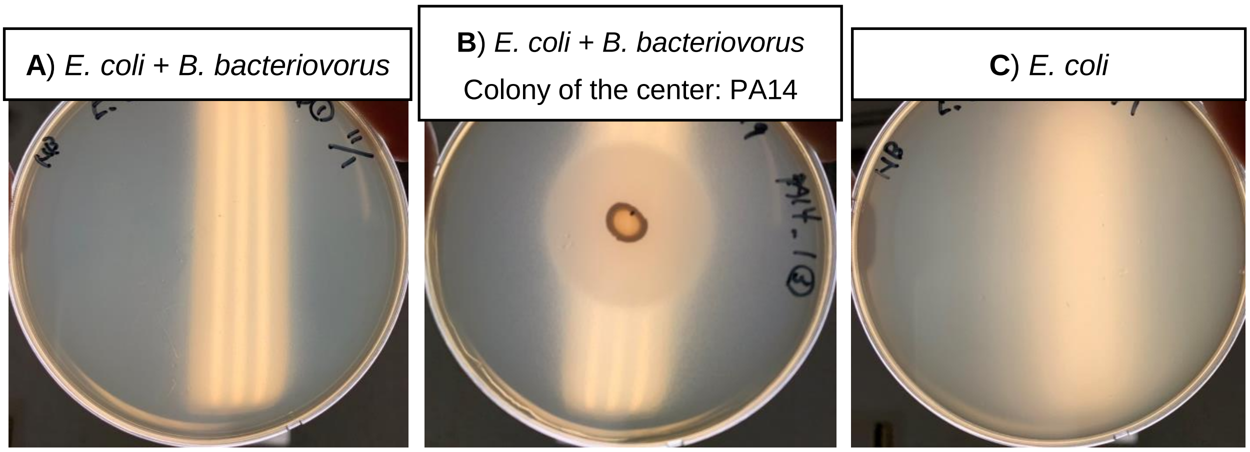

Fig. S1. Plate photographs in the plate assay to investigate the predatory activity of *Bdellovibrio bacteriovorus* and the inhibitory activity in the presence of *Pseudomonas aeruginosa*. The predation plate assays were conducted at 30°C for 4 days by using a mixture of *B. bacteriovorus* 109J and *Escherichia coli* BW25113 in the absence (A) or presence (B) of the colony of *P. aeruginosa* wild type at the center of the plate. In case of using only *E. coli cells* (C), no clear zone was observed due to the absence of *B. bacteriovorus* cells. The presence or absence of a clear zone on the plate should be confirmed by the light transmittance of the fluorescent lamp. The light of fluorescent lamp can pass over the agar plate in the presence of *B. bacteriovorus* (A). Around the colony of *P. aeruginosa*, an inhibition zone (indicating the inhibition of predatory activity) that does not transmit the light of the fluorescent lamp can be confirmed (B). On the other hand, the light of fluorescent lamp does not pass over the agar plate in only *E. coli* cells (C). The fluorescent lamp as background can be clearly seen in the regions of *B. bacteriovorus* growth, but cannot be seen due to turbidity in the regions of *B. bacteriovorus* non-growth (only *E. coli* growth).

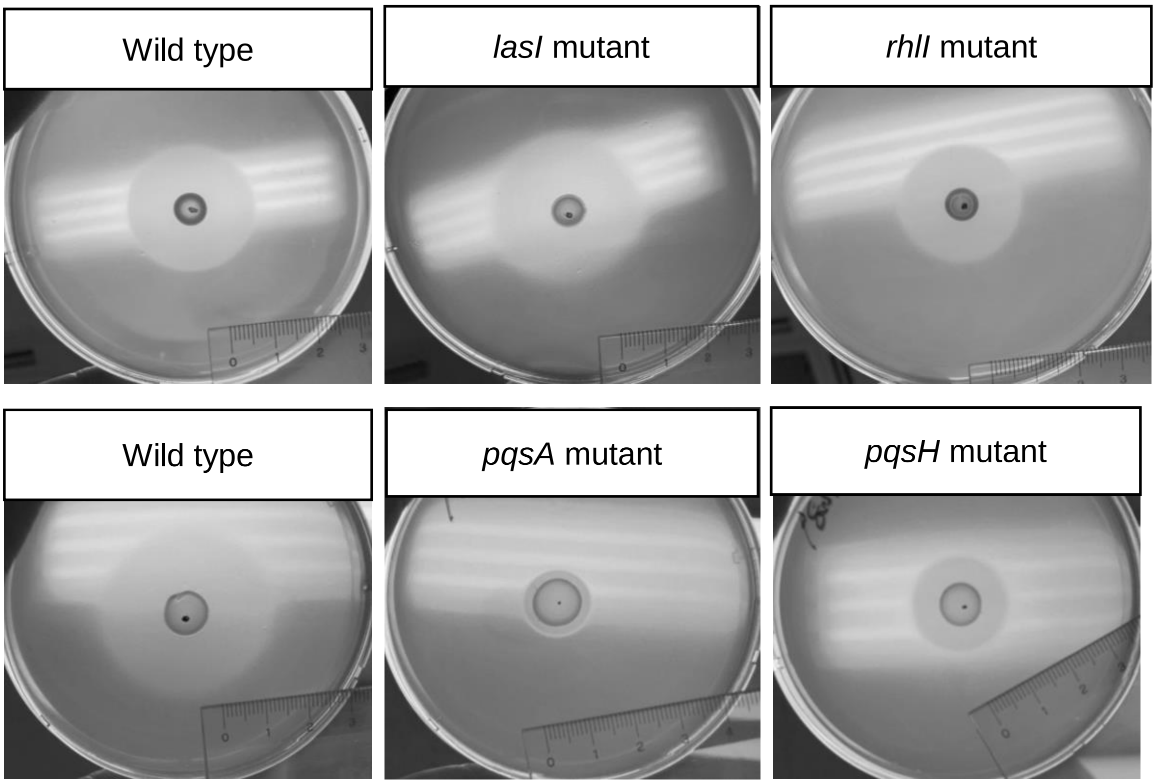

Fig. S2. Plate photographs in the predation plate assays using a mixture of *B. bacteriovorus* and *E. coli* when each cell culture of *P. aeruginosa* wild type, *pqsA* mutant, or *pqsH* mutant was placed at the center of the plate. The predation plate assays were conducted at 30°C for 4 days.

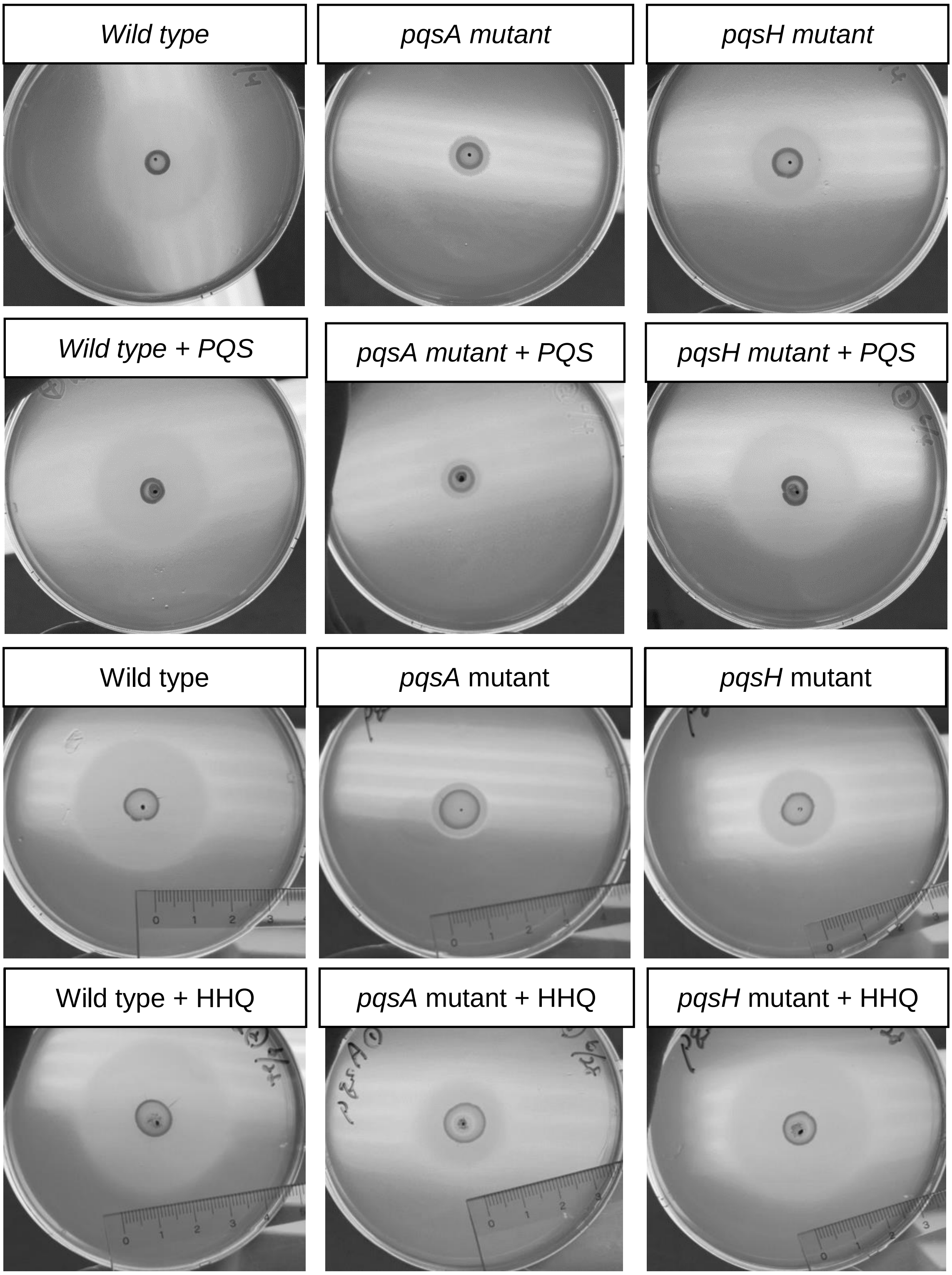

Fig. S3. Plate photographs in the predation plate assays using a mixture of *B. bacteriovorus* and *E. coli* in the presence of *P. aeruginosa* wild type, *pqsA* mutant, or *pqsH* mutant with or without 160 nmol of each quinolone compound (PQS or HHQ). The predation plate assays were conducted at 30°C for 4 days. PQS; *Pseudomonas* quinolone signal, 2-Heptyl-3-hydroxy-4(1H)-quinolone. HHQ; 2-Heptyl-4-quinolone. n = 3.

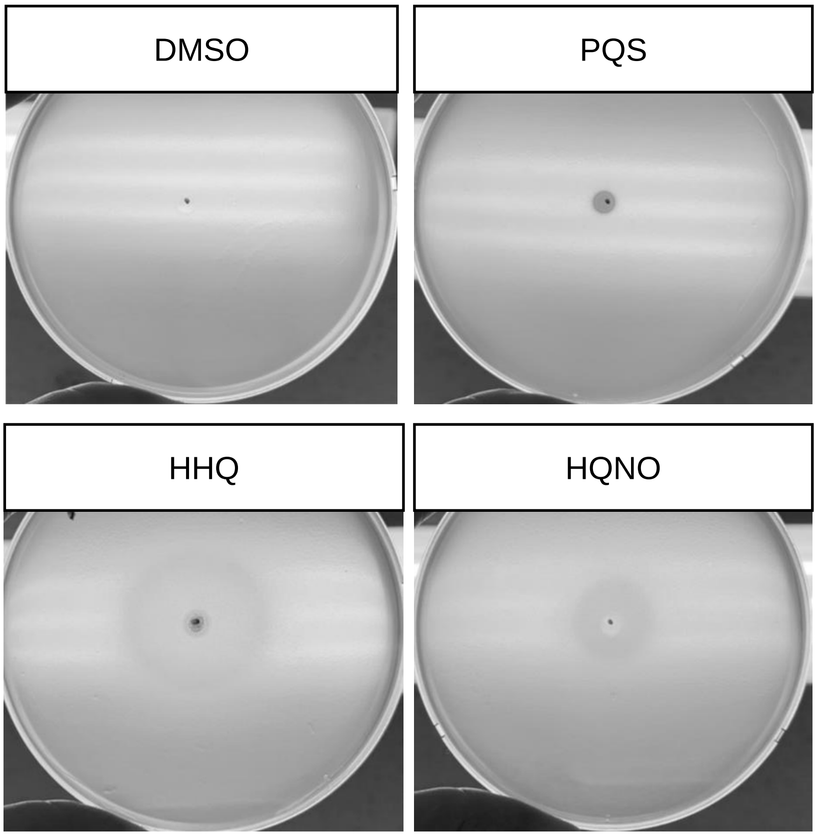

Fig. S4. Effect of PQS, HHQ, or HQNO at a high concentration (160 nmol), or DMSO alone as the control on the predation plate assays using a mixture of *B. bacteriovorus* 109J and *E. coli* BW25113. The photographs indicating the inhibition of predatory activity were also shown in the presence of PQS, HHQ, HQNO (160 nmol), or DMSO alone after the incubation at 30°C for 4 days. PQS; *Pseudomonas* quinolone signal, 2-Heptyl-3-hydroxy-4(1H)-quinolone. HHQ; 2-Heptyl-4-quinolone.

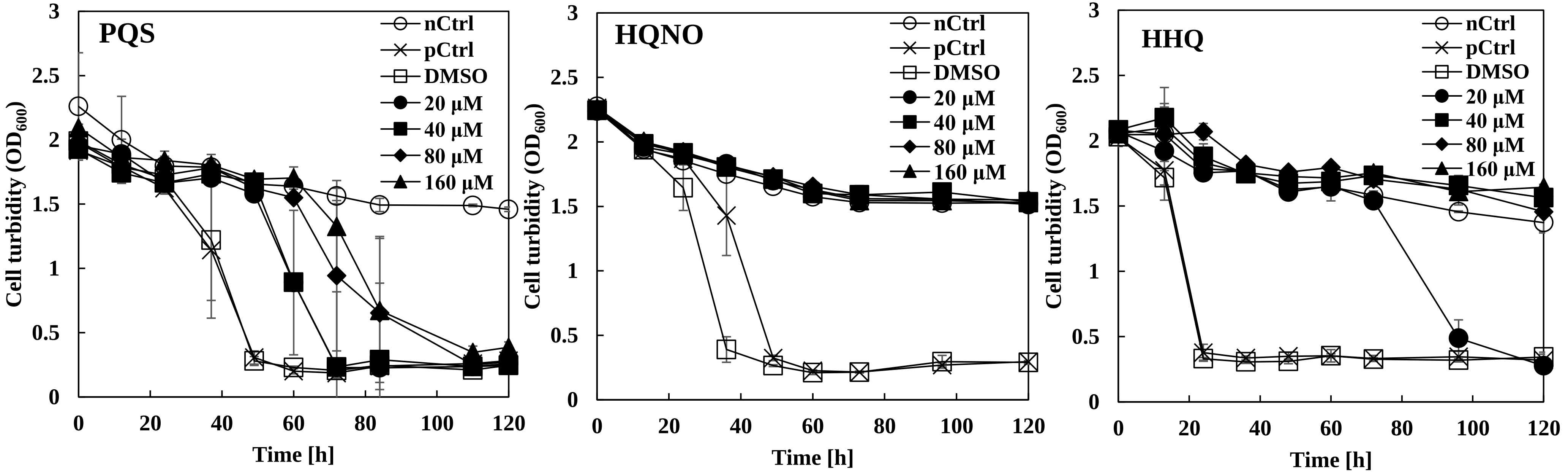

Fig. S5. Effect of each quinolone compound itself at a relatively-high concentration (20 to 160 µM) on the predation liquid assays using a mixture of *B. bacteriovorus* 109J and *E. coli* BW25113. Each cell turbidity of *E. coli* BW25113 ($OD_{600\ nm}$ ~2) was monitored with time after inoculating the cells of *B. bacteriovorus* under different concentrations at 20 µM, 40 µM, 80 µM, or 160 µM of each PQS (left), HQNO (middle), or HHQ (right) compound or DMSO which was used as a solvent for the compounds (as the control). The "nCtrl" means a sample consisting of only the *E. coli* BW25113 cells in HEPES buffer without any compound whereas the "pCtrl" means a mixture of *E. coli* cells and *B. bacteriovorus* cells in HEPES buffer without any compound. PQS; *Pseudomonas* quinolone signal, 2-Heptyl-3-hydroxy-4(1H)-quinolone. HQNO; 2-heptyl-4-quinolinol 1-oxide. HHQ; 2-Heptyl-4-quinolone. n = 3.

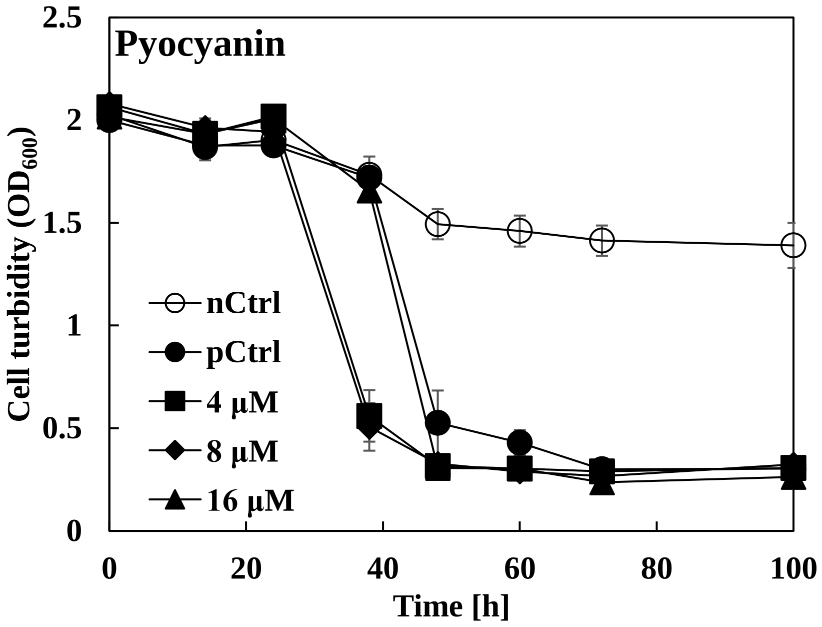

Fig. S6. Effect of pyocyanin, one of the virulence factors on the predation liquid assays using a mixture of *B. bacteriovorus* 109J and *E. coli* BW25113. Each cell turbidity of *E. coli* BW25113 ($OD_{600\ nm}$ ~2) was monitored with time after inoculating the cells of *B. bacteriovorus* under 4 µM, 8 µM, or 16 µM (right) of pyocyanin which was dissolved in sterile H_2O. The "nCtrl" means a sample consisting of only the *E. coli* BW25113 cells in HEPES buffer without any compound whereas the "pCtrl" means a mixture of *E. coli* cells and *B. bacteriovorus* cells in HEPES buffer without any compound. n= 3.

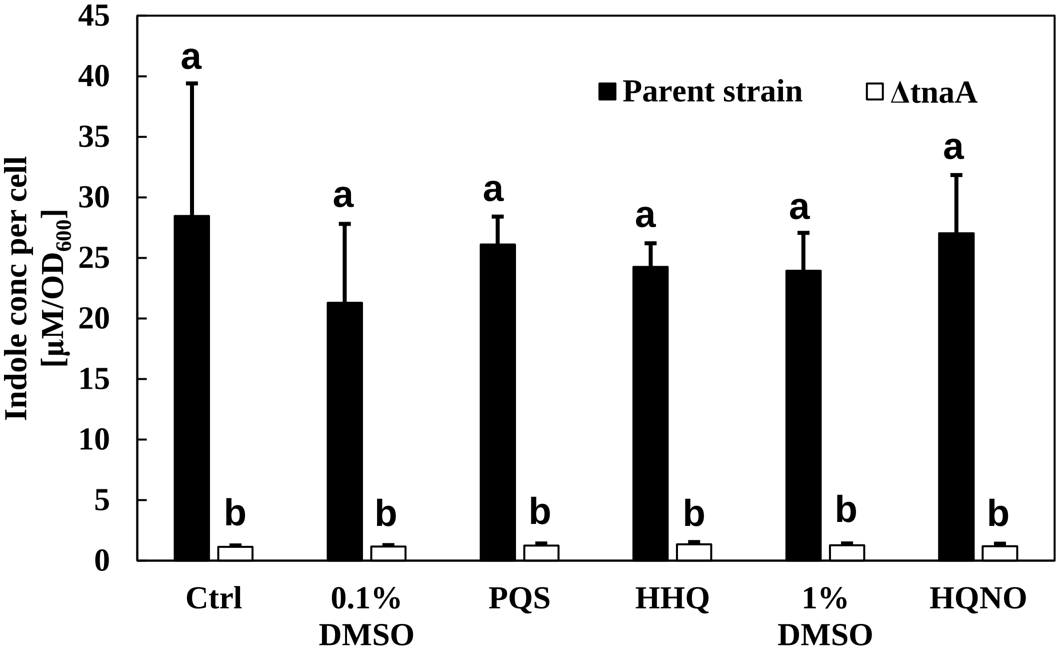

Fig. S7. Indole concentration produced by *E. coli* BW25113 parent strain or *ΔtnaA* under LB medium with or without 80 µM PQS/HHQ/HQNO compound after the incubation at 37°C and 120 rpm for 24 hours. Each stock solution was prepared as 80 mM in DMSO for PQS and HHQ and 8 mM in DMSO for HQNO. Two concentrations of DMSO (0.1% and 1%) were used as a control in the experiments. PQS; *Pseudomonas* quinolone signal, 2-Heptyl-3-hydroxy-4(1H)-quinolone. HHQ; 2-Heptyl-4-quinolone. HQNO; 2-heptyl-4-quinolinol 1-oxide. n = 2. Value among each sample with different letters are significantly different ($p < 0.05$).

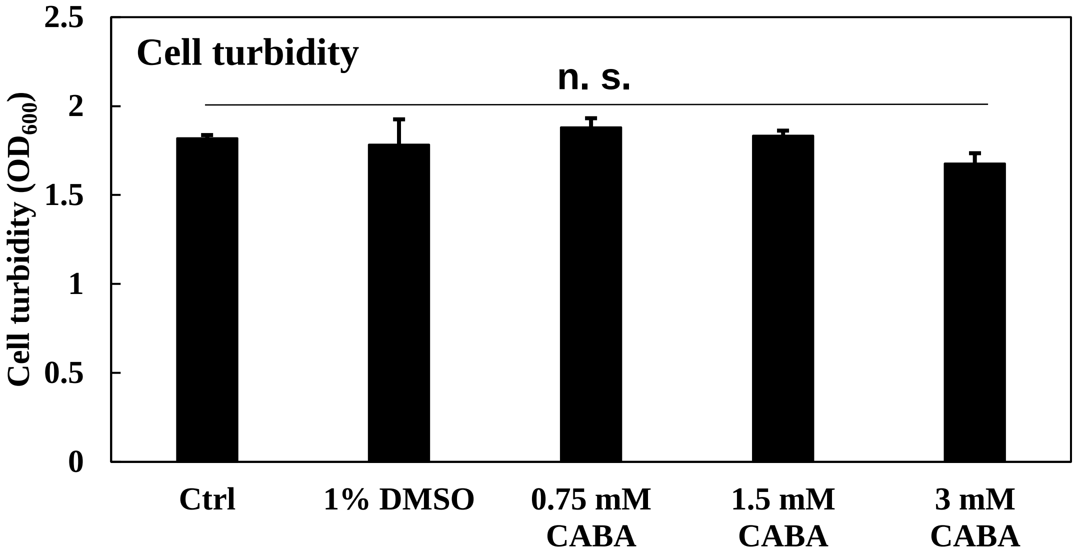

Fig. S8. Effect of 2-amino-6-chlorobenzoic acid (CABA), a *pqs* quorum sensing inhibitor on the growth of *P. aeruginosa*. Cell turbidity of *P. aeruginosa* culture in LB medium with or without 0.75 mM, 1.5 mM, or 3 mM CABA was monitored after the incubation at 37°C and 120 rpm for 10 hours. CABA was dissolved in DMSO; therefore, 1% DMSO was used as a control sample. n = 3. n. s.: no significant.

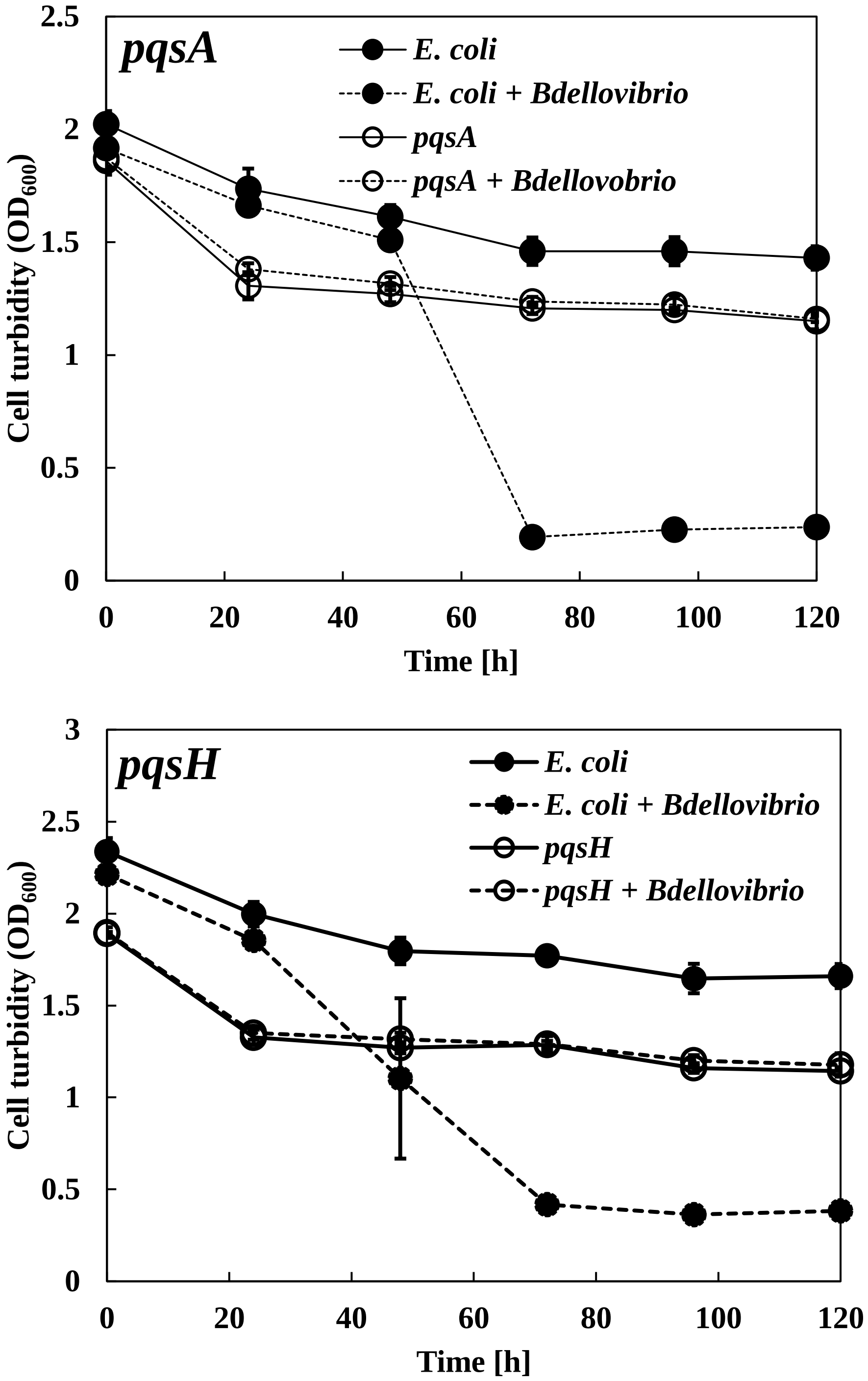

Fig. S9. Effect of no quinolone compounds produced by *P. aeruginosa* each mutant on the predation liquid assay using a mixture of *B. bacteriovorus* with *E. coli* BW25113, or *P. aeuginosa* PA14 *pqsA* (upper) or *pqsH* (bottom) mutant. Each cell turbidity of *E. coli* BW25113, or *P. aeruginosa* PA14 *pqsA* or *pqsH* mutant was monitored with time after inoculating the cells of *B. bacteriovorus*. Each cell suspension (initial cell turbidity was adjusted to be ~2 at OD_{600}) in HEPES buffer was incubated at 30ºC and 120 rpm under an aerobic condition (n = 2). Value among each sample with different letters are significantly different ($p < 0.05$).

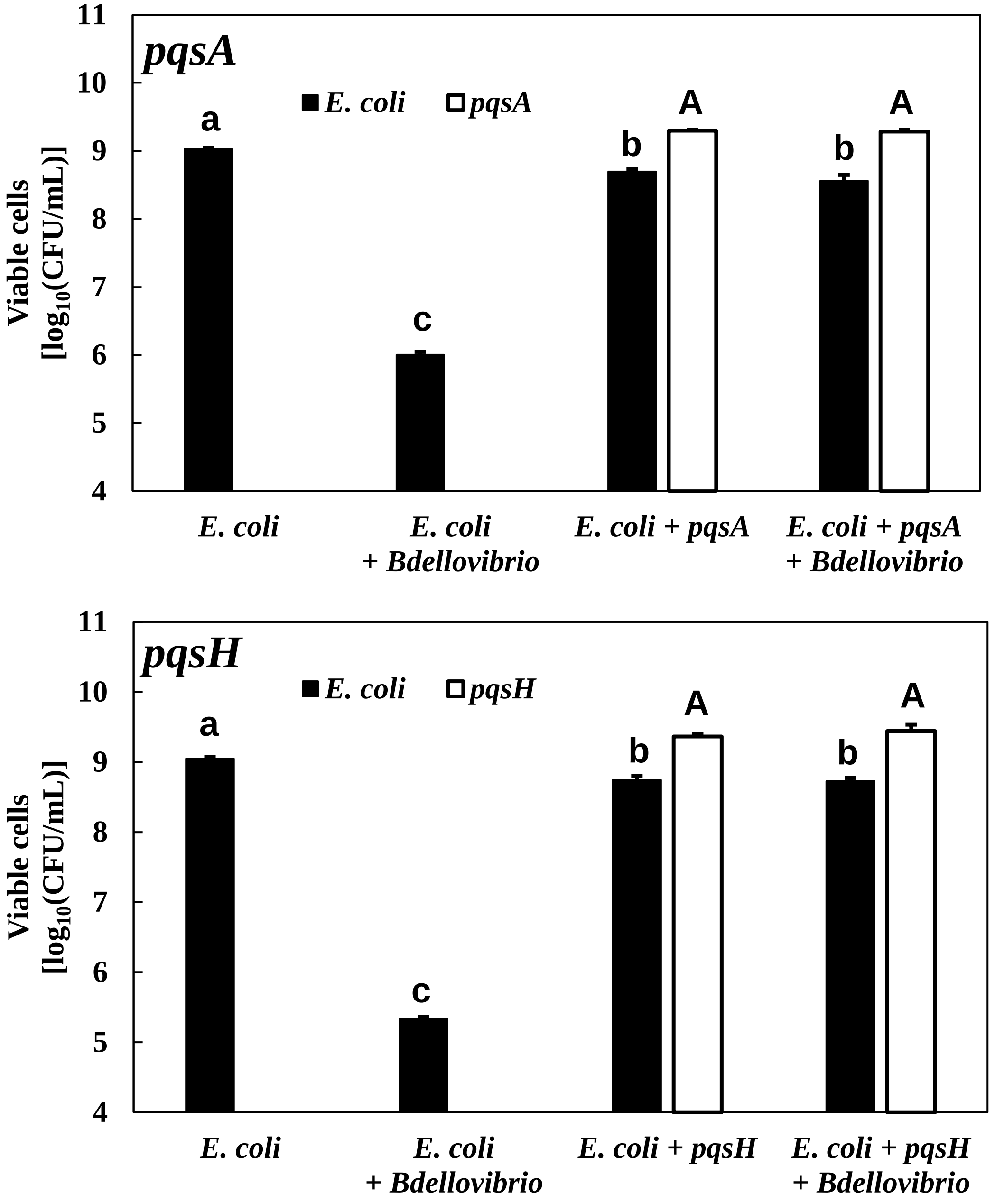

Fig. S10. Inhibition of predatory activity of *Bdellovibrio bacteriovorus* in the presence of *Pseudomonas aeruginosa* PA14 *pqsA* or *pqsH* mutant. Dynamics of viable cells of *Escherichia coli* or/and *P. aeruginosa* PA14 *pqsA* (upper) or *pqsH* (bottom) mutant with or without *B. bacteriovorus* 109J. Each cell suspension (initial cell turbidity was adjusted to be ~2 at OD_{600}) in HEPES buffer was incubated for 120 hours at 30°C and 120 rpm under an aerobic condition (n = 2). Value among each sample with different letters are significantly different ($p < 0.05$).
